# Supplementary material for: The onset of widespread marine red beds and the evolution of ferruginous oceans
Source: Nat Commun. 2017 Aug 30;8:399. doi: 10.1038/s41467-017-00502-x (PMC5577183; doi:10.1038/s41467-017-00502-x)
Supplement: Supplementary file 2 — Supplementary Information [file 41467_2017_502_MOESM2_ESM.pdf]

Description of Supplementary Files

File Name: Supplementary Information
Description: Supplementary Notes, Supplementary Figures, Supplementary Tables and Supplementary References

File Name: Peer Review File

Supplementary Note 1 | The middle Ediacaran MRBs

The first appearance of widespread MRBs in middle Ediacaran successions is of particular importance. Typical examples include the Rainstorm Member of Johnnie Formation in the Death Valley region of western USA[66], the upper Doushantuo Formation in South China[135], the Krol B interval of the Lesser Himalaya, northern India[68, 136], the Chenchinskaya and Alyanchskaya formations in southeast Siberia, Russia[69], the Tikhfist Formation in Morocco [64], and the Lubudi Formation in central Africa[70]. In this study, we examined the MRBs of the Johnnie Formation, the Doushantuo Formation, and the Krol Group.

The pink limestones of the Rainstorm Member (Johnnie Formation) in the Death Valley region rest above the Johnnie oolite and consists of microcrystalline limestone with siltstone and fine-grained sandstone interbeds. They were deposited from shallow subtidal to deep subtidal environments. Ooids, stromatolites, and crystal fans are observed in some of the sections such as in the northern Mesquite Mountains and southern Nopah Range. Carbon isotope analyses have been conducted multiple times for the Rainstorm Member in this region and the data show negative $\delta^{13}C$ values down to –12‰[66, 137, 138], which were correlated with the Shuram excursion. Our carbon isotope analyses of the pink limestones from the northern Mesquite Mountains have $\delta^{13}C$ values of –9.5‰ to –12‰ (Supplementary Table 1 and Fig. 7), consistent with previous studies. The unusually low $\delta^{13}C$ values (down to –12‰) from the pink limestones raised concerns about diagenetic alteration of primary carbon isotope signature, but the consistent spatial and temporal $\delta^{13}C$ pattern[66] and the well-preserved radial fabrics in ooids, stromatolites and crystal fans[67, 139, 140] suggest an overall preservation of primary features.

The pink-red dolostones of the upper Doushantuo Formation in South China is exemplified by the sections in the Yangtze Gorges area. The thinly bedded dolostones are interbedded with shale laminae and are composed of micritic or microcrystalline dolomite suggestive of

deposition from deep subtidal environments below fair-weather wave base. Carbon isotope analyses reveal $\delta^{13}C$ values of –6‰ to –9‰ (Supplementary Table 1 and Fig. 6), which is consistent with the upper Doushantuo negative $\delta^{13}C$ excursion documented from the region[135]. The upper Doushantuo $\delta^{13}C$ excursion has also been correlated to the Shuram excursion[141, 142].

The red beds of the Krol B interval in the Lesser Himalaya, northern India are present in all of the five synclines examined[68, 143]. They are composed of red siltstone, shale and micritic dolostone. The presence of some gypsum pseudomorphs in proximal sections led to the interpretation of intertidal to supratidal environments[143], but our new observations indicate that at least in the distal sections such as in Solan and Korgai synclines, they were likely deposited below the fair-weather wave base. Carbon isotopes of the red dolostones in Krol B vary from –2‰ to –12‰[136], but overall the negative $\delta^{13}C$ shift is apparent at correlative intervals throughout the Krol platform, which has also been correlated with the Shuram excursion[136].

Field and petrographic observations show that the middle Ediacaran MRBs share similar attributes with those of the Triassic and Cretaceous MRBs examined in this study: they are all composed of fine-grained carbonates and shales and have almost no organic matter content or organic-rich interbeds; their red colour is homogenous and does not show patchy staining suggestive of oxidation from reduced iron. Their bulk rock Fe_2O_3 contents are identical (≤1% in carbonates and 1–6% in shales). Petrographic and SEM observations reveal no framboidal or euhedral pyrite precursors. Therefore, we believe that the middle Ediacaran MRBs had the same origin as those of the Triassic and Cretaceous MRBs.

Supplementary Note 2 | Compilation of Phanerozoic MRBs

We document a total of five global MRB intervals from the Phanerozoic, including Cambrian, Late Devonian, Early Triassic, Jurassic and Cretaceous episodes (Supplementary Table 2). Marine red beds are also found in Early Silurian (Telychian) successions in many places including Europe, North America[144, 145] and South China[146], but they are mostly found

distributed around uplifted "old lands" and consist predominately of red sandstone, siltstone and shales without carbonates. The iron source of these Silurian red beds are thought to be of detrital origin[146]. Therefore, we think that these red beds may have been formed differently from the five intervals we have described. However, if future studies indicate that they were formed by similar processes like the ones in Cretaceous and Triassic, the Telychian red beds could be another representative Phanerozoic MRB.

Most of the Phanerozoic MRBs slightly postdate oceanic anoxic events (OAEs), but in a few cases red beds are also found within the interval of oceanic anoxia. One of the examples is the thin red beds within OAE2 in New Zealand[147]. Further study may reveal if this type of red beds records episodic oxidation within a broad anoxic event or a local phenomenon.

Red-pink carbonates of MRBs all have $\delta^{13}C$ values that are lower than temporally adjacent strata, creating "negative" $\delta^{13}C$ excursions (Figs. 2c and 3; Supplementary Figs. 3–7). This is conceivable because oxidation of reduced iron from anoxic waters would inevitably involve oxidation of organic carbon and incorporation of ^{13}C-depleted HCO_3^- during carbonate precipitation, adding ^{13}C-depleted carbon to carbonate. This process may have resulted in negative $\delta^{13}C$ shifts in the range of –0.5‰ to –2‰, as seen in the Phanerozoic MRBs (Figs. 2c and 3; Supplementary Figs. 3–5). The negative $\delta^{13}C$ excursion associated with the middle Ediacaran MRB, or the Shuram $\delta^{13}C$ excursion, however, has a magnitude of ≥ 12‰. While ^{13}C-depleted carbon from oxidation of organic carbon and ^{13}C-depleted HCO_3^- certainly made contributions to the Shuram excursion, the amount of oxidants[148] and reduced carbon source[149] required for the Shuram excursion is enormous and has been highly debated.

Supplementary Note 3 | Debates on the origin of the Shuram $\delta^{13}C$ excursion

The negative $\delta^{13}C$ excursion associated with the middle Ediacaran MRB, or the Shuram excursion, has a magnitude of ≥ 12‰ (ranging from ≥ 4‰ to ≤ –8‰) and a duration of ≥ 5 million years (Myr)[150, 151, 152, 153, 154]. The large magnitude and long duration of this $\delta^{13}C$ excursion make it difficult to interpret using the Phanerozoic carbon cycle models. Early interpretations invoked the upwelling of ^{13}C-depleted deep water[155, 156], but the enormous

amount of ^{13}C-depleted carbon required for accommodating a > 5 Myr $\delta^{13}C$ excursion with a magnitude of ≥ 12‰ is difficult to reconcile. This led to the proposal of a large oceanic dissolved organic carbon (DOC) pool (100–1000 times that of the modern ocean DOC) and perhaps a relatively smaller (than modern) dissolved inorganic carbon pool that was more susceptible to carbon isotope changes[149]. Evidence supporting a large DOC pool came from the decoupled carbonate and organic carbon isotopes prior to and across the Shuram excursion[152, 157]. This hypothesis, however, is challenged by the equally large amount of oxidants required for remineralizing the large DOC pool[148]. Even with the oxidant budget available in the modern surface environments (including atmosphere and ocean) and with an unlimited organic carbon source, it is difficult to support a 12‰ negative $\delta^{13}C$ excursion for more than 3 Myr[148]. In addition, more recent paired carbonate-organic carbon isotope analyses documented decoupled–coupled $\delta^{13}C_{carb}$–$\delta^{13}C_{org}$ patterns from multiple intervals of Ediacaran-Cambrian strata[158, 159, 160], suggesting that even if a large DOC existed in the Precambrian ocean, it was not large enough to buffer the organic carbon isotopes and the evolution of the DOC reservoir was not unidirectional[159, 161].

The shortage of ^{13}C-depleted carbon source or oxidants required for the Shuram excursion led to alternative meteoric[162, 163] and burial[164] diagenetic interpretations. However, both meteoric and burial diagenesis have difficulties of explaining the globally consistent $\delta^{13}C$ excursion across different continents (sedimentary basins) with varying burial history. In addition, most if not all Shuram-age negative $\delta^{13}C$ values are produced in transgressive units above an unconformity where influence from meteoric water should be relatively less significant. The preservation of primary sedimentary structures and fabrics such as crystal fans, radial fabrics of ooids, and microbial laminae in stratigraphic units that host the Shuram excursion also argue against complete recrystallization of carbonate minerals and resetting of isotope signature through burial diagenesis.

A more recent hypothesis invokes authigenic carbonate precipitation in porewater as a possible origin of the Shuram excursion[165]. Due to anoxic bottom waters, authigenic carbonate precipitation in porewaters in Precambrian oceans may have been much more pervasive than in the modern ocean and might be a major ^{13}C-depleted carbon flux. This has

two implications: (1) the cutoff or decline in the global flux of authigenic carbonate would result in a negative $\delta^{13}C$ excursion and (2) the addition of authigenic carbonate into primary marine carbonate would result in localized/regional $\delta^{13}C$ shift. This hypothesis explains some of the spatial variations of the Shuram excursion such as the large isotope gradients[141, 161] and local isotope extremes[166] documented from the Doushantuo Formation in South China, but it cannot explain a global $\delta^{13}C$ excursion with minimum values down to ≤ –12‰ because even a complete cutoff of the authigenic carbonate flux would not result in ocean seawater $\delta^{13}C$ values lower than the riverine (or average crust) $\delta^{13}C$ value of ca. –5‰, unless additional evidence confirm that the Shuram excursion is not globally synchronous.

The debate on the origin of the Shuram excursion (and its correlatives) will continue until better constraints on its magnitude, duration, and spatial variations can be achieved, and our findings by no means solve this debate. However, the coincidence of the Phanerozoic-like, middle Ediacaran MRB and the Shuram excursion does confirm that (1) similar to the negative $\delta^{13}C$ shift associated with the Phanerozoic MRBs, oxidation of organic carbon and incorporation of ^{13}C-depleted HCO_3^- from anoxic waters during carbonate precipitation likely contributed to the Shuram excursion, (2) the larger magnitude of the Shuram excursion may be related to the longer period of anoxia prior to the middle Ediacaran MRB, during which more ^{13}C-depleted carbon may have accumulated through remineralization of organic matter, and (3) iron reduction (using iron oxides as electron acceptors) may have contributed, at least locally, to the heterogeneity of the Shuram excursion.

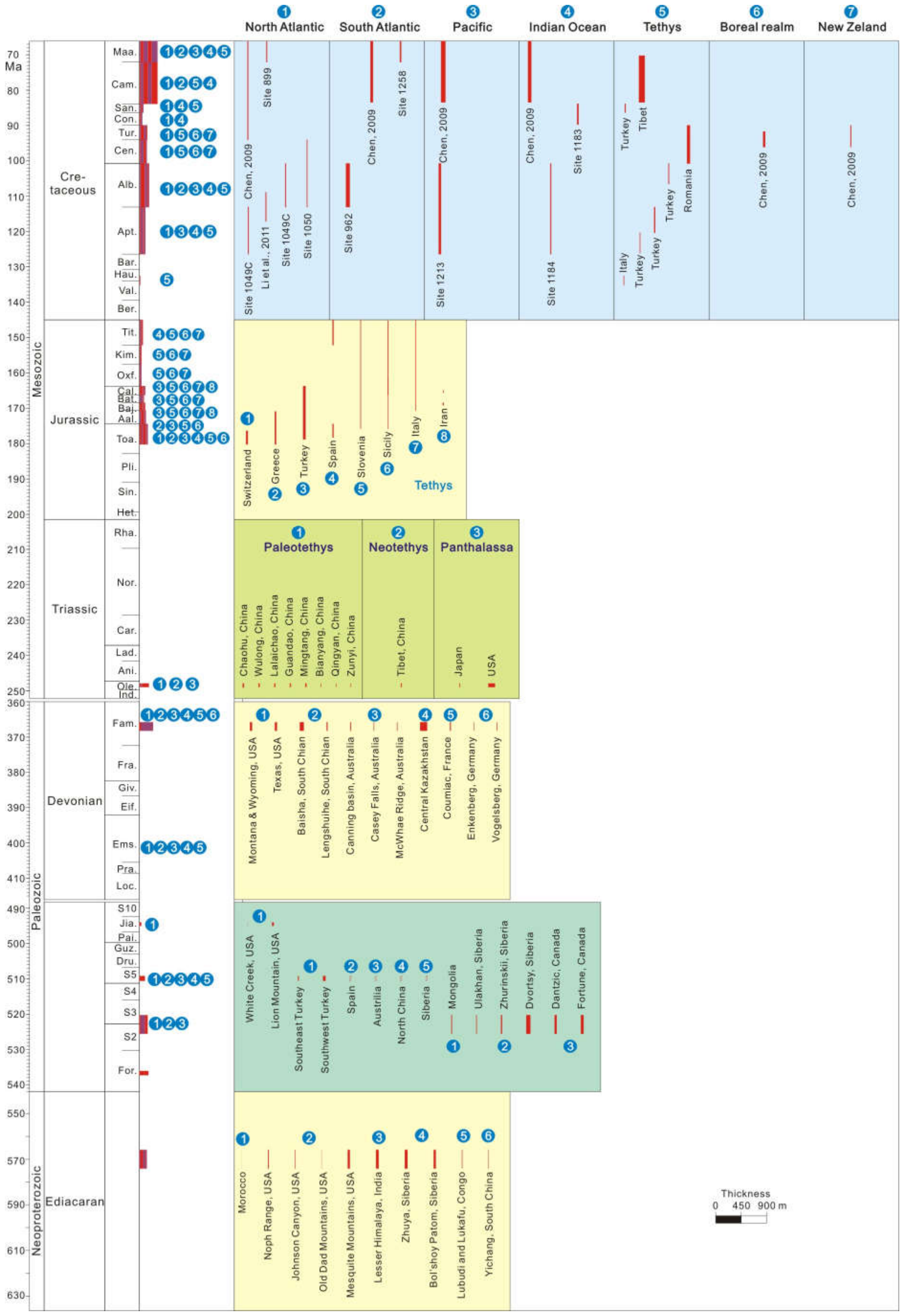

Supplementary Figure 1 | Marine red bed occurrences in the Phanerozoic and Ediacaran. Data are based on Supplementary Table 2.

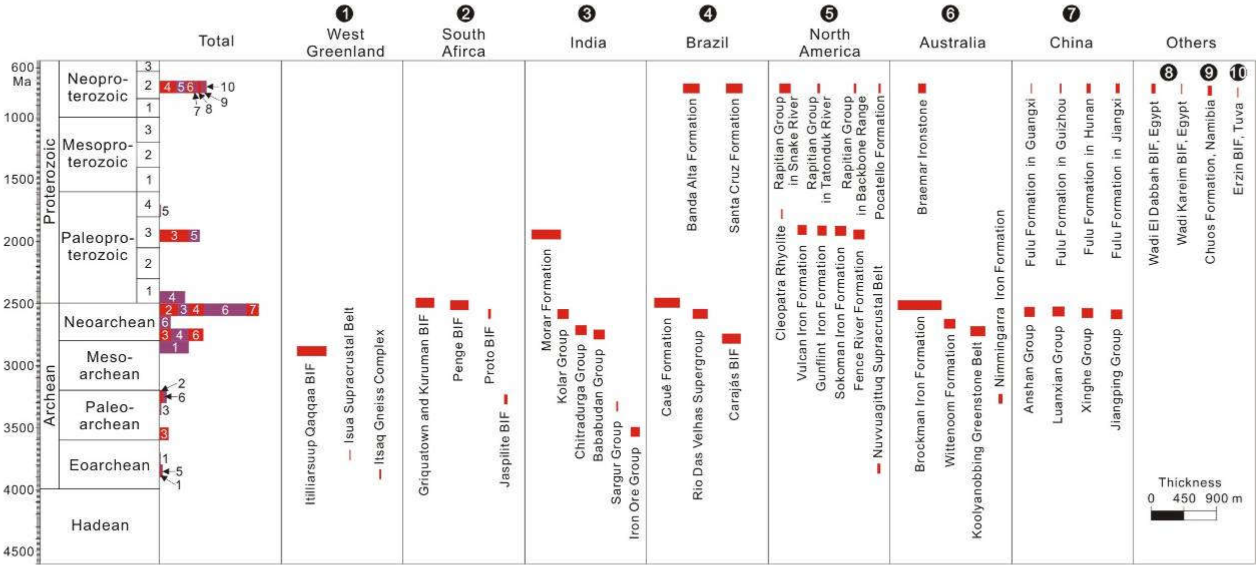

Supplementary Figure 2 | Banded Iron Formation occurrences in the Archean and Proterozoic. Data are based on Supplementary Table 2.

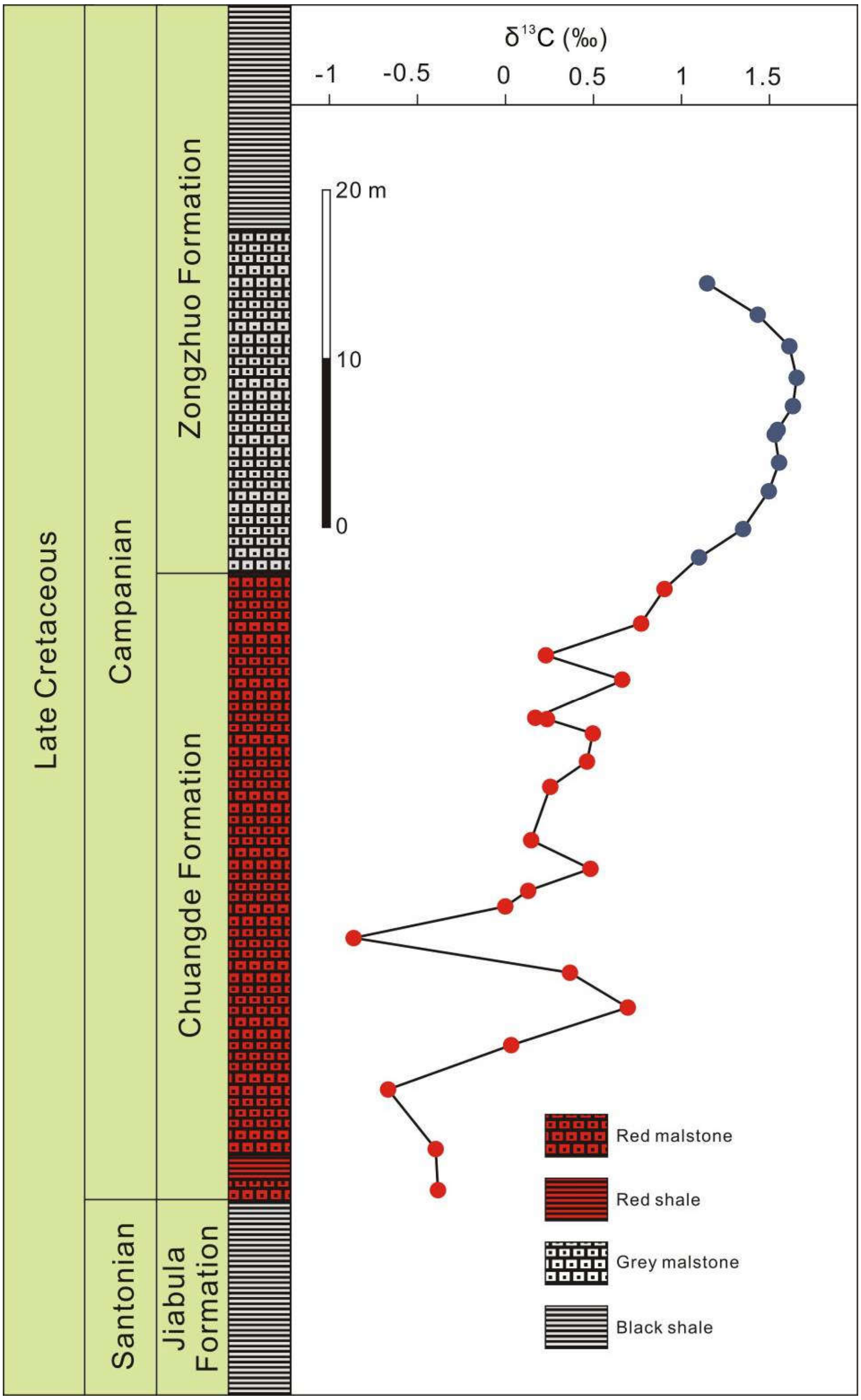

Supplementary Figure 3 | Carbonate $\delta^{13}C$ curve from the Late Cretaceous strata in Chuangde, Tibet, China.

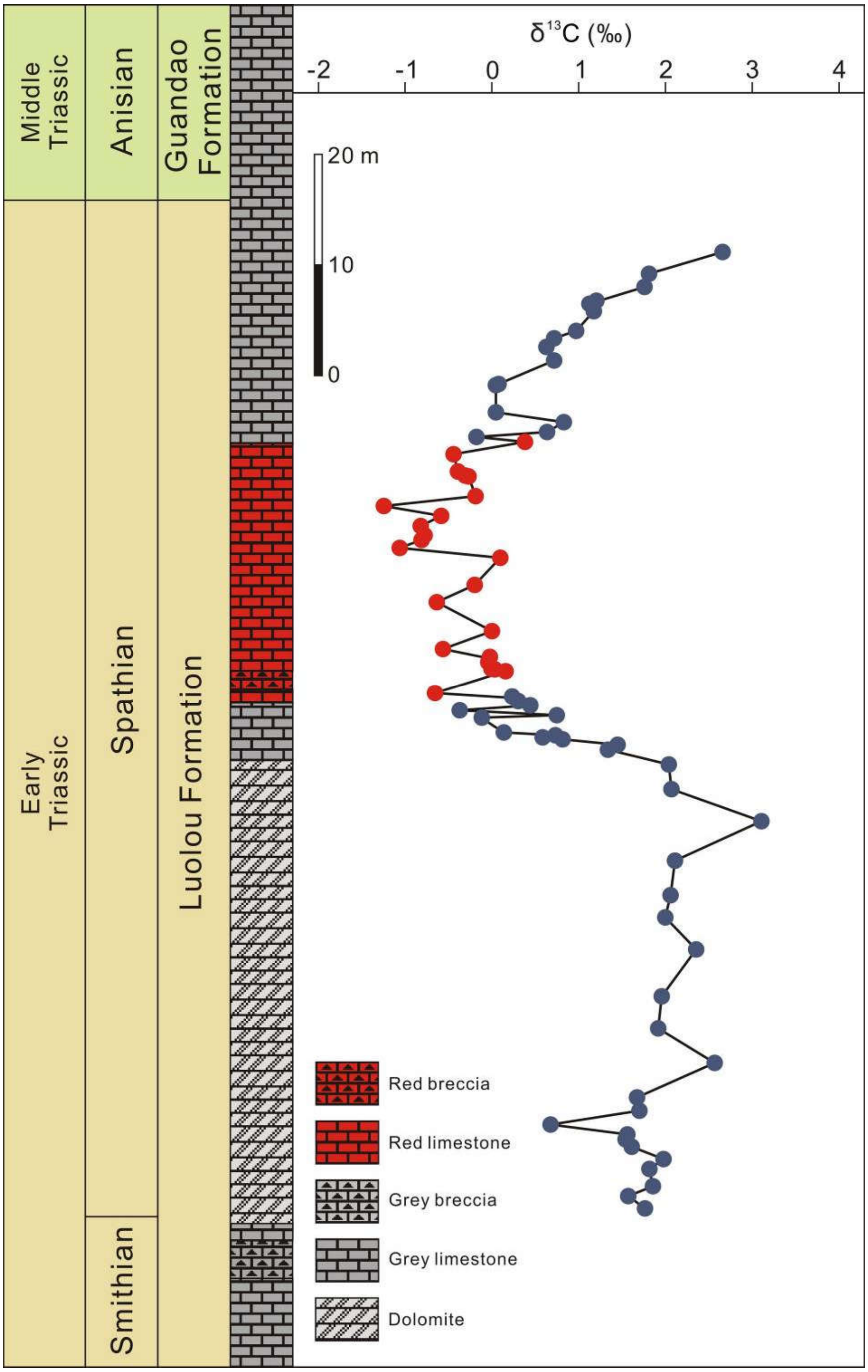

Supplementary Figure 4 | Carbonate $\delta^{13}C$ curve from the Spathian (Early Triassic) in Mingtang, South China.

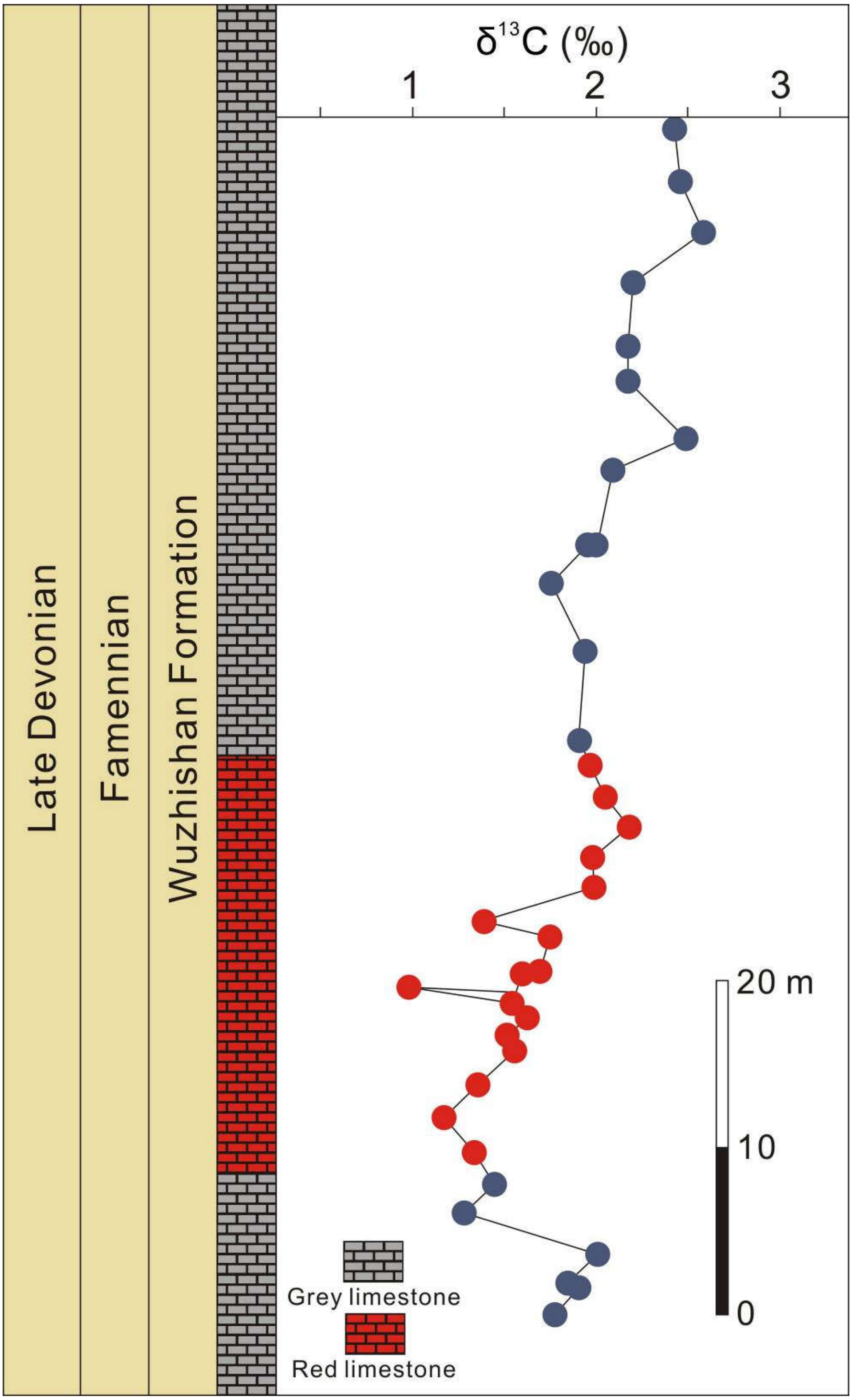

Supplementary Figure 5 | Carbonate $\delta^{13}C$ curve from the Famennian (Late Devonian) strata in Baisha, South China.

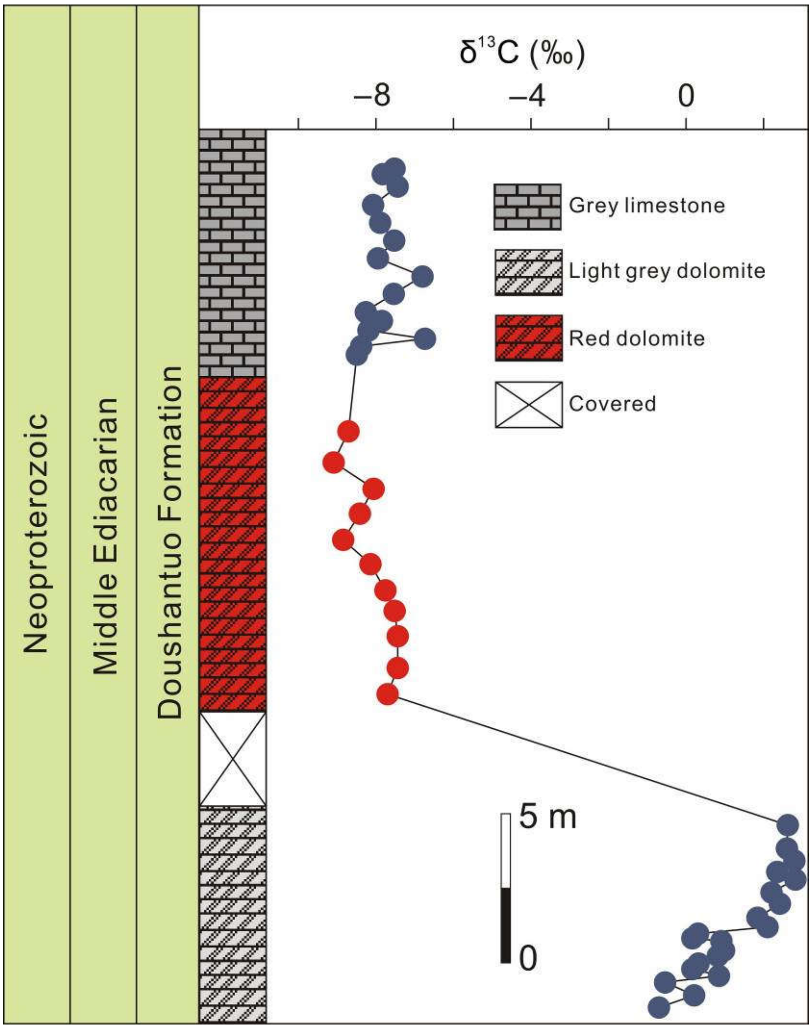

Supplementary Figure 6 | Carbonate $\delta^{13}C$ curves from the middle Ediacaran in Shijiahe, South China.

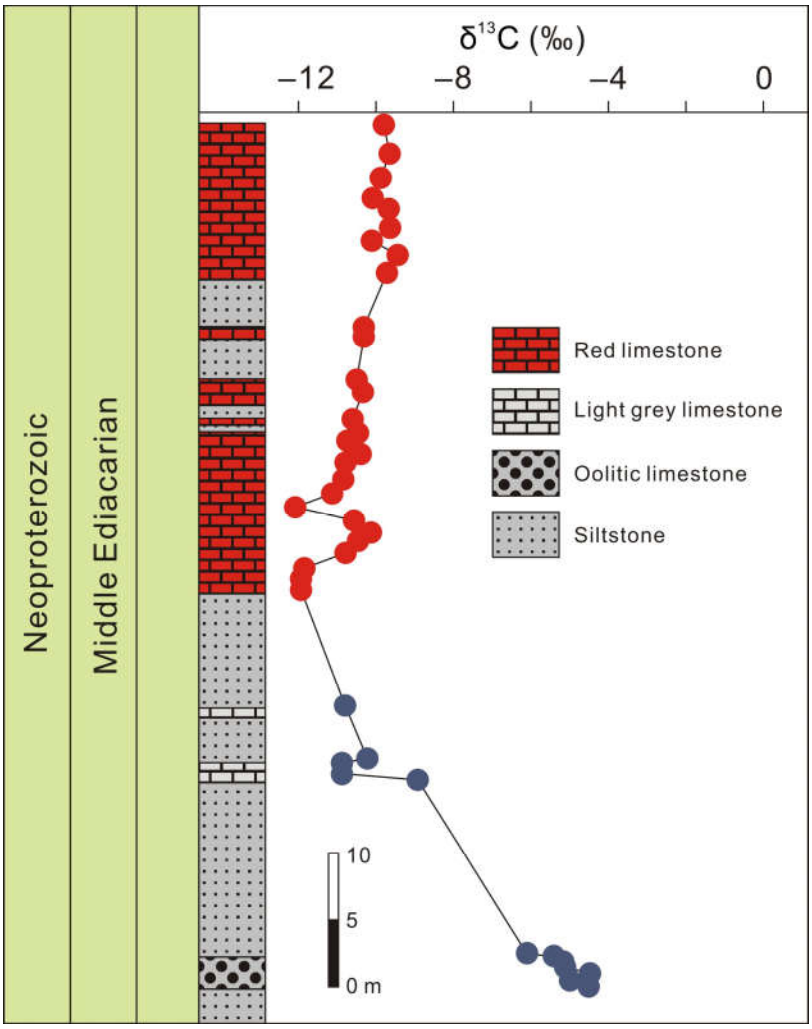

Supplementary Figure 7 | Carbonate δ^{13}C curves from the middle Ediacaran in northern Mesquite Mountains, United States.

Supplementary Table 1 | Carbon and oxygen isotope values of carbonate and iron geochemical data in marine red beds, banded iron formations, and adjacent rocks.

Age (Ma)	Period	Stage	Sample No.	Sample postion (m)	$\delta^{13}C$ (‰)	$\delta^{18}O$ (‰)	Fe_2O_3(%)	Fe^{2+}(%)	Fe^{3+}(%)	Fe^{3+}/Fe^{2+}	Type
~83	Cretaceous	Campanian	CD001	0	-0.38	-13.03					red mudstone
~83	Cretaceous	Campanian	CD002	1.5	-0.4	-12.31	0.53				red mudstone
~83	Cretaceous	Campanian	CD003	3.6	-0.67	-13.43					red mudstone
~83	Cretaceous	Campanian	CD004	3.6	-0.67	-13.42					red mudstone
~83	Cretaceous	Campanian	CD005	5.1	0.03	-13.2					red mudstone
~83	Cretaceous	Campanian	CD006	6.5	0.69	-13	5.2				red mudstone
~83	Cretaceous	Campanian	CD007	7.7	0.37	-13.27					red mudstone
~83	Cretaceous	Campanian	CD008	8.9	-0.86	-15.87					red mudstone
~83	Cretaceous	Campanian	CD009	9.9	0	-14.43	9.23				red mudstone
~83	Cretaceous	Campanian	CD010	10.6	0.13	-11.99					red mudstone
~83	Cretaceous	Campanian	CD011	11.4	0.48	-12.17					red mudstone
~83	Cretaceous	Campanian	CD012	12.4	0.15	-12.22					red mudstone
~83	Cretaceous	Campanian	CD013	14.3	0.25	-11.86					red mudstone
~83	Cretaceous	Campanian	CD014	15.2	0.46	-11.9	6.1				red mudstone
~83	Cretaceous	Campanian	CD015	16.2	0.5	-11.96					red mudstone
~83	Cretaceous	Campanian	CD016	16.7	0.21	-12.4					red mudstone
~83	Cretaceous	Campanian	CD017	16.7	0.17	-12.41					red mudstone
~83	Cretaceous	Campanian	CD018	18.1	0.67	-12.55					red mudstone
~83	Cretaceous	Campanian	CD019	19	0.23	-11.81	2.26				red mudstone
~83	Cretaceous	Campanian	CD020	20.1	0.77	-11.77					red mudstone
~83	Cretaceous	Campanian	CD021	21.3	0.91	-11.95					red mudstone
~83	Cretaceous	Campanian	CD022	22.4	1.1	-11.73					grey mudstone
~83	Cretaceous	Campanian	CD023	23.4	1.35	-11.67	4.15				grey mudstone
~83	Cretaceous	Campanian	CD024	24.8	1.49	-11.65					grey mudstone
~83	Cretaceous	Campanian	CD025	25.8	1.55	-11.7					grey mudstone
~83	Cretaceous	Campanian	CD026	26.7	1.53	-11.79	1.32				grey mudstone
~83	Cretaceous	Campanian	CD027	26.7	1.54	-11.79					grey mudstone
~83	Cretaceous	Campanian	CD028	27.8	1.63	-11.7					grey mudstone
~83	Cretaceous	Campanian	CD029	28.8	1.65	-11.67	1.03				grey mudstone
~83	Cretaceous	Campanian	CD030	29.9	1.61	-11.76					grey mudstone
~83	Cretaceous	Campanian	CD031	31	1.43	-11.91	0.63				grey mudstone
83	Cretaceous	Campanian	CD032	32.1	1.14	-11.91	0.7				grey mudstone
~83	Cretaceous	Campanian	CD033	33.1	0.7	-11.93					grey mudstone
~248	Triassic	Spathian	TL001	11.25	-1.75	-9.69	0.41	0.14	0.14	1.03	grey limestone
~248	Triassic	Spathian	TL002	11.55	-2.32	-12.24	0.32	0.14	0.09	0.62	grey limestone
~248	Triassic	Spathian	TL003	11.95	-1.65	-10.33	0.4	0.16	0.12	0.72	grey limestone
~248	Triassic	Spathian	TL004	12.15	-1.2	-10.16	0.54	0.25	0.13	0.51	grey limestone

~248	Triassic	Spathian	TL005	12.55	-1.46	-10.75	0.45	0.21	0.1	0.48	grey limestone
~248	Triassic	Spathian	TL006	13.4	-1.16	-10.64	0.59	0.22	0.2	0.91	grey limestone
~248	Triassic	Spathian	TL007	13.8	0.02	-11.03	0.68	0.25	0.23	0.9	grey limestone
~248	Triassic	Spathian	TL008	14.4	0.63	-10.57	0.66	0.3	0.16	0.55	grey limestone
~248	Triassic	Spathian	TL009	14.6			3.14	1.78	0.42	0.24	grey limestone
~248	Triassic	Spathian	TL010	14.9	0.72	-12.48	2.62	1.2	0.65	0.54	grey limestone
~248	Triassic	Spathian	TL011	15.02	1.76	-12.35	3.42	1.98	0.41	0.2	grey limestone
~248	Triassic	Spathian	TL012	16.22	-0.45	-13.01	10.19	2.9	4.24	1.46	grey limestone
~248	Triassic	Spathian	TL013	18.62	-0.1	-11.52	1.03	0.29	0.43	1.49	red limestone
~248	Triassic	Spathian	TL014	18.62	-0.09	-11.54	0.79	0.12	0.43	3.53	red limestone
~248	Triassic	Spathian	TL015	19.02	-0.12	-11.71	0.93	0.15	0.51	3.4	red limestone
~248	Triassic	Spathian	TL016	19.54	-0.44	-12.12	0.53	0.11	0.26	2.44	red limestone
~248	Triassic	Spathian	TL017	20.28	-0.46	-11.74	0.56	0.11	0.28	2.56	red limestone
~248	Triassic	Spathian	TL018	21.38	-0.18	-12.11	0.76	0.16	0.37	2.25	red limestone
~248	Triassic	Spathian	TL019	21.68	0.06	-12.09	1.07	0.21	0.54	2.62	red limestone
~248	Triassic	Spathian	TL020	21.98	0.19	-11.89	1.11	0.2	0.57	2.81	red limestone
~248	Triassic	Spathian	TL021	22.48	0.27	-12.15	0.55	0.21	0.18	0.83	grey limestone
~248	Triassic	Spathian	TL022	23.33	0.35	-12.24	0.6	0.26	0.16	0.61	grey limestone
~248	Triassic	Spathian	TL023	23.63	1.52	-12.5	0.77	0.31	0.5	1.61	grey limestone
~248	Triassic	Spathian	TL024	24.33	0.26	-12.66	1.17	0.44	0.82	1.85	grey limestone
~248	Triassic	Spathian	TL025	24.73	0.02	-12.68	0.59	0.24	0.29	1.23	grey limestone
~248	Triassic	Spathian	TL026	25.13	2.36	-11.62	0.76	0.33	0.19	0.58	grey limestone
~248	Triassic	Spathian	TL027	26.63	0.18	-12.71	0.75	0.31	0.57	1.82	grey limestone
~248	Triassic	Spathian	GD001	0.4			0.33	0.14	0.09	0.67	grey limestone
~248	Triassic	Spathian	GD002	1.3			2.34	0.95	0.69	0.72	grey limestone
~248	Triassic	Spathian	GD003	3.8			0.56	0.26	0.13	0.48	grey limestone
~248	Triassic	Spathian	GD004	4.9			0.73	0.37	0.14	0.39	grey limestone
~248	Triassic	Spathian	GD005	6.1			2.71	1.44	0.45	0.31	grey limestone
~248	Triassic	Spathian	GD006	8			0.08	0.05	0.01	0.19	grey limestone
~248	Triassic	Spathian	GD007	9.7			0.17	0.11	0.01	0.11	grey limestone
~248	Triassic	Spathian	GD008	12.4			0.35	0.22	0.02	0.1	grey limestone
~248	Triassic	Spathian	GD009	14.2			1.27	0.5	0.39	0.79	grey limestone
~248	Triassic	Spathian	GD010	39			2.41	1.17	0.51	0.44	grey limestone
~248	Triassic	Spathian	GD011	40.3			2.96	1.17	0.91	0.78	grey limestone
~248	Triassic	Spathian	GD012	41			4.81	1.2	2.17	1.81	red limestone
~248	Triassic	Spathian	GD013	41.5			2.96	0.82	1.26	1.54	red limestone
~248	Triassic	Spathian	GD014	43			3.51	0.87	1.59	1.82	red limestone
~248	Triassic	Spathian	GD015	44			0.12	0.02	0.06	2.51	red limestone
~248	Triassic	Spathian	GD016	45.1			2.43	0.88	0.82	0.92	red limestone
~248	Triassic	Spathian	GD017	45.9			2.92	0.66	1.38	2.08	red limestone
~248	Triassic	Spathian	GD018	46.2			2.12	0.47	1.02	2.16	red limestone
~248	Triassic	Spathian	GD019	47			2.71	0.64	1.26	1.99	red limestone

~248	Triassic	Spathian	GD020	47.4			1.73	0.44	0.78	1.79	red limestone
~248	Triassic	Spathian	GD021	48			0.35	0.16	0.08	0.53	red limestone
~248	Triassic	Spathian	GD022	48.9			2.69	0.61	1.27	2.08	red limestone
~248	Triassic	Spathian	GD023	49.8			1.84	0.44	0.86	1.96	red limestone
~248	Triassic	Spathian	GD024	50.3			2.36	0.54	1.11	2.08	red limestone
~248	Triassic	Spathian	GD025	51.3			1.58	0.39	0.71	1.8	red limestone
~248	Triassic	Spathian	GD026	52.5			1.43	0.34	0.66	1.96	red limestone
~248	Triassic	Spathian	GD027	53.3			1.01	0.19	0.52	2.72	red limestone
~248	Triassic	Spathian	GD028	53.7			1.31	0.36	0.56	1.56	red limestone
~248	Triassic	Spathian	GD029	54.15			0.41	0.09	0.19	2.08	red limestone
~248	Triassic	Spathian	GD030	54.95			1.66	0.48	0.68	1.43	red limestone
~248	Triassic	Spathian	GD031	55.75			1.73	0.42	0.79	1.9	red limestone
~248	Triassic	Spathian	GD032	56.35			0.22	0.1	0.06	0.56	red limestone
~248	Triassic	Spathian	GD033	57.55			1.7	0.51	0.69	1.36	red limestone
~248	Triassic	Spathian	GD034	60.6			0.08	0.03	0.02	0.76	grey limestone
~248	Triassic	Spathian	GD035	64.3			0.06	0.02	0.02	0.84	grey limestone
~248	Triassic	Spathian	GD036	66.3			0.28	0.1	0.1	0.96	grey limestone
~248	Triassic	Spathian	GD037	67.6			0.38	0.2	0.07	0.34	grey limestone
~248	Triassic	Spathian	GD038	68.4			0.14	0.04	0.06	1.5	grey limestone
~248	Triassic	Spathian	MT001	14.4	1.77	-5.32					light grey dolomite
~248	Triassic	Spathian	MT002	15.5	1.57	-9.96					light grey dolomite
~248	Triassic	Spathian	MT003	15.5	1.57	-10.32					light grey dolomite
~248	Triassic	Spathian	MT004	16.3	1.85	-5.6					light grey dolomite
~248	Triassic	Spathian	MT005	17.8	1.81	-6.26					light grey dolomite
~248	Triassic	Spathian	MT006	18.6	1.98	-7.24					light grey dolomite
~248	Triassic	Spathian	MT007	21	1.61	-8.12					light grey dolomite
~248	Triassic	Spathian	MT008	22	1.6	-8.07					light grey dolomite
~248	Triassic	Spathian	MT009	22.5	1.56	-7.47					light grey dolomite
~248	Triassic	Spathian	MT010	23.3	0.67	-8.77					light grey dolomite
~248	Triassic	Spathian	MT011	24.5	1.7	-7.6					light grey dolomite
~248	Triassic	Spathian	MT012	25.7	1.67	-8.09					light grey dolomite
~248	Triassic	Spathian	MT013	29.8	2.57	-4.34					light grey dolomite
~248	Triassic	Spathian	MT014	32	1.91	-4.32					light grey dolomite
~248	Triassic	Spathian	MT015	34.8	1.95	-5.77					light grey dolomite
~248	Triassic	Spathian	MT016	39	2.36	-4.44					light grey dolomite
~248	Triassic	Spathian	MT017	42	2	-4.9					light grey dolomite
~248	Triassic	Spathian	MT018	44	2.05	-9.03					light grey dolomite
~248	Triassic	Spathian	MT019	47.2	2.11	-5.43					light grey dolomite
~248	Triassic	Spathian	MT020	50.6	3.11	-4.73					light grey dolomite
~248	Triassic	Spathian	MT021	53.5	2.06	-4.95					light grey dolomite
~248	Triassic	Spathian	MT022	55.8	2.04	-5.69					light grey dolomite
~248	Triassic	Spathian	MT023	57.2	1.33	-3.93					grey limestone

~248	Triassic	Spathian	MT024	57.6	1.45	-3.45					grey limestone
~248	Triassic	Spathian	MT025	51.8	0.59	-7					grey limestone
~248	Triassic	Spathian	MT026	58.1	0.81	-7.56					grey limestone
~248	Triassic	Spathian	MT027	58.4	0.71	-7.81					grey limestone
~248	Triassic	Spathian	MT028	58.7	0.14	-10.69					grey limestone
~248	Triassic	Spathian	MT029	60	-0.12	-10.91					grey limestone
~248	Triassic	Spathian	MT030	60.4	0.75	-5.63					grey limestone
~248	Triassic	Spathian	MT031	60.8	-0.37	-8.15					grey limestone
~248	Triassic	Spathian	MT032	61.3	0.44	-3.71					grey limestone
~248	Triassic	Spathian	MT033	61.3	0.44	-3.52					grey limestone
~248	Triassic	Spathian	MT034	61.8	0.28	-4.29					grey limestone
~248	Triassic	Spathian	MT035	62	0.24	-4.33					grey limestone
~248	Triassic	Spathian	MT036	62.4	-0.65	-8.71					red limestone
~248	Triassic	Spathian	MT037	64.2	0.16	-5.34					red limestone
~248	Triassic	Spathian	MT038	64.6	-0.01	-6.49					red limestone
~248	Triassic	Spathian	MT039	65	0.02	-6.16					red limestone
~248	Triassic	Spathian	MT040	65.5	-0.05	-5.96					red limestone
~248	Triassic	Spathian	MT041	65.8	-0.03	-5.88					red limestone
~248	Triassic	Spathian	MT042	66.4	-0.56	-6.69					red limestone
~248	Triassic	Spathian	MT043	68	0.01	-4.41					red limestone
~248	Triassic	Spathian	MT044	70.5	-0.64	-6.49					red limestone
~248	Triassic	Spathian	MT045	72	-0.2	-5.03					red limestone
~248	Triassic	Spathian	MT046	74.5	0.1	-3.57					red limestone
~248	Triassic	Spathian	MT047	74.5	0.09	-3.56					red limestone
~248	Triassic	Spathian	MT048	75.5	-1.06	-8.12					red limestone
~248	Triassic	Spathian	MT049	76	-0.79	-8.2					red limestone
~248	Triassic	Spathian	MT050	76.5	-0.78	-5.79					red limestone
~248	Triassic	Spathian	MT051	77.4	-0.81	-6.46					red limestone
~248	Triassic	Spathian	MT052	78.3	-0.58	-4.77					red limestone
~248	Triassic	Spathian	MT053	79.4	-1.25	-6.89					red limestone
~248	Triassic	Spathian	MT054	80	-0.19	-3.91					red limestone
~248	Triassic	Spathian	MT055	82	-0.27	-4.45					red limestone
~248	Triassic	Spathian	MT056	82.2	-0.29	-4.43					red limestone
~248	Triassic	Spathian	MT057	82.5	-0.39	-4.85					red limestone
~248	Triassic	Spathian	MT058	84	-0.44	-5.24					red limestone
~248	Triassic	Spathian	MT059	85	0.38	-4.78					red limestone
~248	Triassic	Spathian	MT060	85.5	-0.18	-5.69					grey limestone
~248	Triassic	Spathian	MT061	86	0.63	-4.04					grey limestone
~248	Triassic	Spathian	MT062	86.8	0.83	-5.57					grey limestone
~248	Triassic	Spathian	MT063	87.8	0.04	-5.11					grey limestone
~248	Triassic	Spathian	MT064	90	0.04	-5.49					grey limestone
~248	Triassic	Spathian	MT065	90.4	0.06	-5.58					grey limestone

~248	Triassic	Spathian	MT066	92.4	0.72	-4.72					grey limestone
~248	Triassic	Spathian	MT067	93.6	0.62	-2.23					grey limestone
~248	Triassic	Spathian	MT068	94.3	0.71	-2.17					grey limestone
~248	Triassic	Spathian	MT069	95	0.97	-2.95					grey limestone
~248	Triassic	Spathian	MT070	96.8	1.17	-2.37					grey limestone
~248	Triassic	Spathian	MT071	97.5	1.16	-3.47					grey limestone
~248	Triassic	Spathian	MT072	98	1.14	-3.35					grey limestone
~248	Triassic	Spathian	MT073	99	1.77	-2.02					grey limestone
~248	Triassic	Spathian	MT074	100.4	1.8	-2.92					grey limestone
~248	Triassic	Spathian	MT075	102.3	2.65	-1.93					grey limestone
~370	Devonian	Famennian	BS001	118	1.89	-5.71					grey limestone
~370	Devonian	Famennian	BS002	120	2	-6.56	2.81				grey limestone
~370	Devonian	Famennian	BS003	122	1.3	-4.95					grey limestone
~370	Devonian	Famennian	BS004	124	1.44	-5.48					grey limestone
~370	Devonian	Famennian	BS005	126	1.33	-5.93					red limestone
~370	Devonian	Famennian	BS006	128	1.17	-6					red limestone
~370	Devonian	Famennian	BS007	130	1.35	-5.78					red limestone
~370	Devonian	Famennian	BS008	130	1.34	-5.9					red limestone
~370	Devonian	Famennian	BS009	132	1.55	-5.61	0.3				red limestone
~370	Devonian	Famennian	BS010	133	1.51	-5.68					red limestone
~370	Devonian	Famennian	BS011	134	1.63	-5.87					red limestone
~370	Devonian	Famennian	BS012	135	1.55	-5.87	0.86				red limestone
~370	Devonian	Famennian	BS013	136	0.98	-6.06					red limestone
~370	Devonian	Famennian	BS014	136	1.58	-6.26					red limestone
~370	Devonian	Famennian	BS015	137	1.69	-6.25					red limestone
~370	Devonian	Famennian	BS016	139	1.75	-4.59					red limestone
~370	Devonian	Famennian	BS017	140	1.37	-8.81	0.3				red limestone
~370	Devonian	Famennian	BS018	142	1.98	-6.36					red limestone
~370	Devonian	Famennian	BS019	144	1.98	-6.48					red limestone
~370	Devonian	Famennian	BS020	145.5	2.21	-5.92					red limestone
~370	Devonian	Famennian	BS021	147.5	2.05	-6.08					red limestone
~370	Devonian	Famennian	BS022	149.5	1.96	-6.64	0.65				red limestone
~370	Devonian	Famennian	BS023	151.5	1.9	-6.39					grey limestone
~370	Devonian	Famennian	BS024	156.5	1.95	-6.49					grey limestone
~370	Devonian	Famennian	BS025	161	1.73	-6.15					grey limestone
~370	Devonian	Famennian	BS026	163	1.97	-6.9	0.31				grey limestone
~370	Devonian	Famennian	BS027	163	2.01	-6.82					grey limestone
~370	Devonian	Famennian	BS028	167.5	2.09	-6.5					grey limestone
~370	Devonian	Famennian	BS029	169.5	2.49	-6.4					grey limestone
~370	Devonian	Famennian	BS030	173	2.17	-5.69					grey limestone
~370	Devonian	Famennian	BS031	175	2.18	-6.25					grey limestone
~370	Devonian	Famennian	BS032	179	2.2	-5.69					grey limestone

~370	Devonian	Famennian	BS033	182	2.59	-5.42					grey limestone
~370	Devonian	Famennian	BS034	185	2.46	-5.5					grey limestone
~370	Devonian	Famennian	BS035	188	2.41	-4.98					grey limestone
~580	Ediacaran		NM001	64.8	-9.83	-10.09					red dolomite
~580	Ediacaran		NM002	63	-9.7	-10.2					red dolomite
~580	Ediacaran		NM003	61	-9.9	-9.65					red dolomite
~580	Ediacaran		NM004	59.5	-10.1	-8.63					red dolomite
~580	Ediacaran		NM005	58.5	-9.73	-9.65					red dolomite
~580	Ediacaran		NM006	57	-9.72	-9.06					red dolomite
~580	Ediacaran		NM007	56	-10.06	-8.61					red dolomite
~580	Ediacaran		NM008	55	-9.52	-7.58					red dolomite
~580	Ediacaran		NM009	53.5	-9.76	-9.73					red dolomite
~580	Ediacaran		NM010	50	-10.32	-9.56					red dolomite
~580	Ediacaran		NM011	49	-10.31	-9.09					red dolomite
~580	Ediacaran		NM012	46	-10.54	-9.09					red dolomite
~580	Ediacaran		NM013	45	-10.38	-10.35					red dolomite
~580	Ediacaran		NM014	43	-10.63	-7.65					red dolomite
~580	Ediacaran		NM015	42	-10.53	-9.71					red dolomite
~580	Ediacaran		NM016	41	-10.78	-8.3					red dolomite
~580	Ediacaran		NM017	40	-10.42	-7.73					red dolomite
~580	Ediacaran		NM018	39.5	-10.78	-7.72					red dolomite
~580	Ediacaran		NM019	38	-10.88	-8.34					red dolomite
~580	Ediacaran		NM020	37	-11.09	-6.68					red dolomite
~580	Ediacaran		NM021	36	-12.12	-7.75					red dolomite
~580	Ediacaran		NM022	35	-10.67	-7.95					red dolomite
~580	Ediacaran		NM023	34	-10.15	-8.35					red dolomite
~580	Ediacaran		NM024	33.5	-10.44	-8.01					red dolomite
~580	Ediacaran		NM025	33.5	-10.55	-8.12					red dolomite
~580	Ediacaran		NM026	32.5	-10.82	-6.07					red dolomite
~580	Ediacaran		NM027	31.5	-11.92	-8.11					red dolomite
~580	Ediacaran		NM028	30.3	-11.99	-8.09					red dolomite
~580	Ediacaran		NM029	29.5	-11.94	-8.26					red dolomite
~580	Ediacaran		NM030	21	-10.84	-10.92					light grey dolomite
~580	Ediacaran		NM031	17	-10.25	-8.17					light grey dolomite
~580	Ediacaran		NM032	16.5	-10.92	-7.51					light grey dolomite
~580	Ediacaran		NM033	16	-10.87	-8.82					light grey dolomite
~580	Ediacaran		NM034	15.5	-8.94	-7.72					light grey dolomite
~580	Ediacaran		NM035	2.4	-6.1	-8.14					oolitic dolomite
~580	Ediacaran		NM036	2.1	-5.36	-7.58					oolitic dolomite
~580	Ediacaran		NM037	1.8	-5.12	-8.25					oolitic dolomite
~580	Ediacaran		NM038	1.5	-5.26	-8.34					oolitic dolomite
~580	Ediacaran		NM039	1.2	-5.1	-9.97					oolitic dolomite

~580	Ediacaran		NM040	0.8	-4.47	-8.2						oolitic dolomite
~580	Ediacaran		NM041	0.8	-4.49	-8.28						oolitic dolomite
~580	Ediacaran		NM042	0.5	-4.63	-9.93						oolitic dolomite
~580	Ediacaran		NM043	0.3	-4.96	-10.16						oolitic dolomite
~580	Ediacaran		NM044	0	-4.55	-7.85						oolitic dolomite
~580	Ediacaran		SJH001	0.5	-0.69	-6.3						light grey dolomite
~580	Ediacaran		SJH002	1	0.22	-4.81						light grey dolomite
~580	Ediacaran		SJH003	1.3	-0.53	-4.87						light grey dolomite
~580	Ediacaran		SJH004	1.7	0.9	-2.64						light grey dolomite
~580	Ediacaran		SJH005	1.9	0.17	-2.38						light grey dolomite
~580	Ediacaran		SJH006	2	0.34	-2.03						light grey dolomite
~580	Ediacaran		SJH007	2.2	0.8	-1.51						light grey dolomite
~580	Ediacaran		SJH008	2.5	0.95	-2.43						light grey dolomite
~580	Ediacaran		SJH009	2.8	0.92	-2.48						light grey dolomite
~580	Ediacaran		SJH010	2.9	0.16	-4.28						light grey dolomite
~580	Ediacaran		SJH011	3	0.31	-3.42						light grey dolomite
~580	Ediacaran		SJH012	3.2	2.1	-2.68						light grey dolomite
~580	Ediacaran		SJH013	3.6	1.84	-2.96						light grey dolomite
~580	Ediacaran		SJH014	4	2.43	-1.92						light grey dolomite
~580	Ediacaran		SJH015	4.4	2.22	-2.4						light grey dolomite
~580	Ediacaran		SJH016	4.8	2.82	-2.17						light grey dolomite
~580	Ediacaran		SJH017	5.2	2.36	-3.65	0.58					light grey dolomite
~580	Ediacaran		SJH018	5.5	2.83	-2.45						light grey dolomite
~580	Ediacaran		SJH019	5.7	2.79	-2.59						light grey dolomite
~580	Ediacaran		SJH020	5.9	2.61	-3.43	0.44					light grey dolomite
~580	Ediacaran		SJH021	5.9	2.62	-2.96						light grey dolomite
~580	Ediacaran		SJH022	6.7	2.63	-4.31	0.27					light grey dolomite
~580	Ediacaran		SJH023	11	-7.64	-6.87						red dolomite
~580	Ediacaran		SJH024	11.8	-7.38	-7.37						red dolomite
~580	Ediacaran		SJH025	13	-7.38	-7.33						red dolomite
~580	Ediacaran		SJH026	13.8	-7.48	-7.21	3.99					red dolomite
~580	Ediacaran		SJH027	14.6	-7.7	-7.83	3.66					red dolomite
~580	Ediacaran		SJH028	15.3	-8.09	-7.75						red dolomite
~580	Ediacaran		SJH029	16.2	-8.78	-8.42	2.46					red dolomite
~580	Ediacaran		SJH030	17.1	-8.36	-7.83						red dolomite
~580	Ediacaran		SJH031	18	-8.01	-7.95	0.22					red dolomite
~580	Ediacaran		SJH032	18.8	-9.03	-8.4						red dolomite
~580	Ediacaran		SJH033	19.8	-8.65	-8.6	1.98					red dolomite
~580	Ediacaran		SJH034	22.4	-8.45	-9.14						grey limestone
~580	Ediacaran		SJH035	22.8	-8.32	-8.29	0.49					grey limestone
~580	Ediacaran		SJH036	23	-6.69	-5.52						grey limestone
~580	Ediacaran		SJH037	23.2	-8.14	-9						grey limestone

~580	Ediacaran		SJH038	23.6	-7.8	-7.13	3.19				grey limestone
~580	Ediacaran		SJH039	23.8	-8.23	-9.31					grey limestone
~580	Ediacaran		SJH040	24.5	-7.49	-7.42					grey limestone
~580	Ediacaran		SJH041	25.1	-6.76	-9.28					grey limestone
~580	Ediacaran		SJH042	25.7	-7.9	-8.94					grey limestone
~580	Ediacaran		SJH043	26.2	-7.48	-8.16					grey limestone
~580	Ediacaran		SJH044	26.8	-7.84	-8.17					grey limestone
~580	Ediacaran		SJH045	27.5	-8.02	-8.06					grey limestone
~580	Ediacaran		SJH046	28	-7.38	-7.38					grey limestone
~580	Ediacaran		SJH047	28.2	-7.78	-8.54					grey limestone
~580	Ediacaran		SJH048	28.5	-7.49	-8.49					grey limestone
~580	Ediacaran		SJH049	28.8	-7.8	-3.11					grey limestone
~730	Tonian		SYB001	4	-6.01	-23.13	10.02				marble
~730	Tonian		SYB002	12	-6.5	-22.89					marble
~730	Tonian		SYB003	19.5	-6.31	-22.93					marble
~730	Tonian		SYB004	21	-5.76	-22.3	5.65				marble
~730	Tonian		SYB005	28	-6.81	-21.79					marble
~730	Tonian		SYB006	36	-11.06	-19.28	22.45				banded iron formation
~730	Tonian		SYB007	43.6	-8.62	-19.73	8.06				marble
~730	Tonian		SYB008	52.5	-11.94	-19.22	1.67				marble
~730	Tonian		SYB009	55	-8.51	-17.97	9.17				marble
~730	Tonian		SYB010	57.5	-6.52	-24.16					marble
~730	Tonian		SYB011	60.6	-5.83	-24.96	1.67				marble
~730	Tonian		SYB012	63.5	-6.41	-24.52					marble
~730	Tonian		SYB013	67.5	-4.78	-16.45					marble
~730	Tonian		SYB014	71.5	-4.72	-16.29					marble
~730	Tonian		SYB015	77	-3.78	-11.41	4.72				marble
~730	Tonian		SYB016	83.5	-1.86	-13.86					marble
~2500	Siderian		KJG001	170	-2.98	-19.65	14.4				calcareous schist
~2500	Siderian		KJG002	160	-3.06	-19.8					calcareous schist
~2500	Siderian		KJG003	150	-3.46	-10.86					calcareous schist
~2500	Siderian		KJG004	145	0.13	-5.21	16.23				calcareous schist
~2500	Siderian		KJG005	140	-3.03	-19.85					calcareous schist
~2500	Siderian		KJG006	130	-5.37	-9.37					banded iron formation
~2500	Siderian		KJG007	128	-2.62	-7.88					banded iron formation
~2500	Siderian		KJG008	126	-4.43	-8.48					banded iron formation
~2500	Siderian		KJG009	122	-5.32	-8.39	23.81				banded iron formation
~2500	Siderian		KJG010	120	-3.83	-8.66					banded iron formation
~2500	Siderian		KJG011	118	-3.68	-18.48					banded iron formation
~2500	Siderian		KJG012	118	-3.68	-18.47	41.98				banded iron formation
~2500	Siderian		KJG013	116	-3.94	-8.56					banded iron formation

~2500	Siderian		KJG014	114	-4.06	-17.14						banded iron formation
~2500	Siderian		KJG015	108	-3.45	-16.91						banded iron formation
~2500	Siderian		KJG016	107	-3.69	-16.93	41.92					banded iron formation
~2500	Siderian		KJG017	106	-3.58	-16.67						banded iron formation
~2500	Siderian		KJG018	104.5	-1.39	-8.33						banded iron formation
~2500	Siderian		KJG019	102.5	-3.39	-9.6	37.02					banded iron formation
~2500	Siderian		KJG020	101	-3.58	-17.47						banded iron formation
~2500	Siderian		KJG021	100.3	-3.42	-16.88						banded iron formation
~2500	Siderian		KJG022	100.3	-3.32	-16.81						banded iron formation
~2500	Siderian		KJG023	100.3	-3.67	-17.25	30.02					banded iron formation
~2500	Siderian		KJG024	99.8	-2.02	-18.59						calcareous schist
~2500	Siderian		KJG025	99.3	-2.21	-16.81						calcareous schist
~2500	Siderian		KJG026	99.3	-2.5	-16.93						calcareous schist
~2500	Siderian		KJG027	99	-1.98	-16.46						calcareous schist
~2500	Siderian		KJG028	98.8	-2.05	-18.52	18.05					calcareous schist
~2500	Siderian		KJG029	98.8	-1.97	-18.59						calcareous schist
~2500	Siderian		KJG030	98.8	-1.81	-18.32						calcareous schist
~2500	Siderian		KJG031	80	-1.9	-19.81						calcareous schist
~2500	Siderian		KJG032	77	-2.08	-19.61						calcareous schist
~2500	Siderian		KJG033	77	-1.94	-19.72						calcareous schist
~2500	Siderian		KJG034	65	-1.27	-19.64	18.77					calcareous schist
~2500	Siderian		KJG035	60	-1.46	-19.12						calcareous schist
~2500	Siderian		KJG036	50	-2.15	-19.19						calcareous schist
~2500	Siderian		KJG037	40	-1.72	-19.76	17.68					calcareous schist

Supplementary Table 2 | MRBs and BIFss in the Phanerozoic and Precambrian.

Age (Ma)	Period/Stage	Formation	Lithology	Thickness	Locations	References of MRB and BIF	Paleolatitude	References of Paleolatitude
70	Maastrichtian		claystone	10 m	ODP-Leg 149-Site 899, North Atlantic	1	N0-30	2
70	Maastrichtian		claystone	10 m	ODP-Leg 171B-Site 1049C, North Atlantic	1	N0-30	2
70	Maastrichtian		limestone	20 m	ODP-Leg 207-Site 1258, South Atlantic	1	S0-30	2
80	Campanian				Globally distribution	3	N30-60	2
80	Campanian				Globally distribution	3	N0-30	2
80	Campanian				Globally distribution	3	S0-30	2
80	Campanian				Globally distribution	3	S30-60	2
80	Campanian	Chuangde Formation	shale, limestone	25 m	Gyangze Chuangde section, Tibet	4	S0-30	2
83	Santonian-Lower Campanian	Chuangde Formation	limestone, shale	30 m	Chuangde section, Tibet	This study	S0-30	2
83.6-66	Campanian-Maastrichtian		claystone	23 m	ODP-Leg 171B-Site 1050, North Atlantic	1	N0-30	2
83.6-66	Campanian-Maastrichtian		limestone, claystone	30-80 m	South Atlantic	5	S30-60	2
83.6-66	Campanian-Maastrichtian		limestone, claystone	0.7-150 m	Pacific	5	S0-30	2
83.6-66	Campanian-Maastrichtian		claystone	13-115 m	Indian Ocean	5	S30-60	2
83.6-70	Campanian-early Maastrichtian	Chuangde Formation	limestone, shale	125 m	Tianba section, Tibet	6	S0-30	2
84-72.1	upper Santonian-Campanian		limestone, claystone	10-600 m	Tethys	5	S0-30	2
84	late Santonian	Dicarinella asymetrica zone	limestone	5 m	Çavuşdere section, Turkey	7	N0-30	2
84	late Santonian	Dicarinella asymetrica zone	limestone	7 m	Değirmenözü section, Turkey	7	N0-30	2
84	late Santonian	Dicarinella asymetrica zone	limestone	6 m	Samsaçavuş section, Turkey	7	N0-30	2
84	late Santonian	Dicarinella asymetrica zone	limestone	3 m	Mudurnu section, Turkey	7	N0-30	2
84	late Santonian	Dicarinella asymetrica zone	limestone	9 m	Göynük-Sünnet section, Turkey	7	N0-30	2
84	late Santonian	Dicarinella asymetrica zone	limestone	13 m	İsmailler section, Turkey	7	N0-30	2
89.8-83.6	Coniacian-Santonian		limestone	42 m	ODP-Leg 192-Site 1183, Indian Ocean	1	S30-60	2

93.5-65	Turonian-early Paleocene	Scaglia Rossa	limestone	260 m	Bottaccione section, Italy	8	N0-30	2
93.9-66	Turonian-Maastrichtian		claystone	30-60 m	North Atlantic	5	N0-30	2
93.9-83.6	Turonian-Santonian		claystone	45 m	ODP-Leg 210-Site 1276, North Atlantic	1	N0-30	2
93.9-86.3	Turonian-Coniacian		claystone	19 m	ODP-Leg 171B-Site 1050, North Atlantic	1	N0-30	2
95-86.3	late Cenomanian-Coniacian		claystone	10-20 m	New Zeland	5	S60-90	2
95-90	late Cenomanian, middle Turonian	Sohlde Formation	limestone	35 m	Boreal realm	5	N30-60	2
97-95	Cenomanian	Mazak Formation	shale		Outer Western Carpathians, Czech	9	N30-60	2
93	Turonian	Helvetotruncana helvetica Zone	mudstone	5 m	Buchberg, Switzerland	10	N30-60	2
95	late Cenomanian	*Wheitenella arche ocretcea* Zone	shale	2 m	Çavuşdere section, Turkey	7	N0-30	2
95	late Cenomanian	*Wheitenella arche ocretcea* Zone	shale	2 m	Göynük-Sünnet section, Turkey	7	N0-30	2
95	late Cenomanian	*Wheitenella arche ocretcea* Zone	shale	3 m	İsmailler section, Turkey	7	N0-30	2
100-88	Cenomanian-Turonian	Bota-Botita Formation	shale	120 m	Audia Nappe, Romania	11	N30-60	2
100-88	Cenomanian-Turonian	Carnu-Siclau Formation	shale	60 m	Tarcau Nappe, Romania	11	N30-60	2
100-88	Cenomanian-Turonian		claystone	19.3 m	DSDP-Leg 11-Site 105, Atlantic Ocean	1	N30-60	2
101-95	late Albian-Cenomanian	Skalski Marl Member	Mudstone, shale		Pieniny Klippen Basin, Poland	9	N30-60	2
101-99	late Albian	Untere Bunte Schiefer	shale		Rhenodanubian Flysch, Austria	9	N30-60	2
101-99	late Albian				East Carpathians, Romania	9	N30-60	2
101-99	late Albian	Fatu La Formation	limestone		Zanskar Himalaya	9	S0-30	2
105	mid-Cretaceous				Globally distribution	3	N0-30	2
105	mid-Cretaceous				Globally distribution	3	S0-30	2
105	mid-Cretaceous				Globally distribution	3	S30-60	2
105	late Albian	*Biticinella breggie nsis* Zone	shale	8 m	Samsaçavuş section, Turkey	7	N0-30	2
105	late Albian	*Biticinella breggie nsis* Zone	shale	3 m	Mudurnu section, Turkey	7	N0-30	2
108-100	Albian	Red Chalk Formation	chalk		Northeastern England	9	N30-60	2

110	Albian		claystone	10 m	ODP-Leg 171B-Site 1049C, North Atlantic	1	N0-30	2
110	Albian		claystone	65 m	ODP-Leg 159-Site 962, South Atlantic	1	S30-60	2
113-93.9	Albian-Cenomanian		claystone	13 m	ODP-Leg 171B-Site 1050, North Atlantic	1	N0-30	2
113	late Aptian-early Albian	*Paraticinella eub ejaouaensis* Zone	mudstone	10 m	North Atlantic	12	N0-30	2
115	late Aptian	*Planomalina cheniourensis* Zone	limestone	3 m	Soğukçam section, Turkey	7	N0-30	2
115	late Aptian	*Planomalina cheniourensis* Zone	limestone	15 m	Değirmenözü section, Turkey	7	N0-30	2
115	late Aptian	*Planomalina cheniourensis* Zone	limestone	1 m	Mudurnu section, Turkey	7	N0-30	2
115	late Aptian	*Globigerinelloids algerianus* Zone	limestone	4 m	Sünnetgölü section, Turkey	7	N0-30	2
116-112	Aptian	Schrambach Formation	limestone		North Calcareous Alps, Austria	9	N30-60	2
116-112	Aptian	Medoveevskaya Formation	mudstone		Caucasus	9	N60-90	2
118-108	Aptian-Albian		claystone		Atlantic	9	N0-30	2
118-108	Aptian-Albian		claystone		Atlantic	9	S0-30	2
118-108	Aptian-Albian		claystone		Atlantic	9	S30-60	2
120	early Aptian	*Leupoldina cabri* Zone	shale	3 m	Samsaçavuş section, Turkey	7	N0-30	2
120	Aptian		claystone	10 m	ODP-Leg 171B-Site 1049C, North Atlantic	1	N0-30	2
120	Aptian		claystone	5 m	ODP-Leg 192-Site 1187, Indian Ocean	1	S30-60	2
125-100	Aptian and Albian	Marnea Fucoidi	limestone, claystone	~30 m	Piobbico Core, Italy	9	N0-30	2
125-100.5	Aptian-Albian		claystone	78 m	ODP-Leg 198-Site 1213, Pacific Ocean	5	N0-30	2
125-100.5	Aptian-Albian		claystone	60 m	ODP-Leg 198-Site 1214, Pacific Ocean	5	S0-30	2
125-100.5	Aptian-Albian		claystone	22 m	ODP-Leg 192-Site 1184, Indian Ocean	1	S30-60	2
125-100.5	Aptian-Albian		claystone	3.3 m	DSDP-Leg 41-Site 367, Atlantic Ocean	1	N0-30	2
133	late Valanginian-early Hauterivian	Rosso Ammonitico	limestone	5 m	Trento Plateau, Italy	13	N0-30	2

		Puezzese						
150	Tithonian		limestone	15 m	Subbetic Cordillera, Spain	14	N0-30	2
163.5-145	Oxfordian-Tithonian		limestone	9-13 m	Betic Cordillera, Spain	15	N0-30	2
165	middle Callovian	Dalichai Formation	limestone	6 m	east Alborz, Iran	16	N30-60	2
165	Callovian	Tabanos Formation	limestone, mudstone		Neuquén Basin, Argentina	17	S30-60	2
166.1-139.6	Callovian-late Berriasian	Rosso Ammonitico	limestone	35 m	Monte Inici, Sicily	18	N0-30	2
166.1-145	Callovian-Tithonian	Ammonitico Rosso Veronese	limestone		Trento Plateau, Italy	19	N0-30	2
169-145	late Bajocian-Tithonian	Rosso Ammonitico Veronese	limestone	< 30 m	Verona, Italy	20	N0-30	2
169-145	late Bajocian-Tithonian	Rosso Ammonitico Inferiore	limestone	18 m	Asiago, Italy	20	N0-30	2
168.3	late Bajocian	Dalichai Formation	limestone	15 m	east Alborz, Iran	16	N30-60	2
170.3-145	Bajocian-Tithonian	Rosso Ammonitico Veronese	limestone	28 m	Northeastern Italy	21	N0-30	2
170.3-145	Bajocian-Tithonian	Rosso Ammonitico Veronese	limestone	12 m	Luznic Lake area, Slovenia	22	N30-60	2
~170	Bajocian	Rosso Ammonitico Veronese	limestone	~3 m	Triglav Lake Valley, Slovenia	22	N30-60	2
~170	Bajocian		shale		Central Japan	23	N30-60	2
174.1-145.0	late Toarcian-Tithonian	Rosso Ammonitico Veronese	limestone	16 m	Julian Alps, Slovenia	22	N30-60	2
174.1-145.0	late Toarcian-Tithonian	Rosso Ammonitico	limestone	16 m	Western Sicily, Italy	24	N0-30	2
174.1-157.3	Toarcian-Oxfordian	Rosso Ammonitico	limestone	4 m	MonteKumeta, Sicily	25	N0-30	2
175-163.5	late Toarcian-Callovian	Rosso Ammonitico	limestone	~50 m	Ankara, Turkey	26	N30-60	2
175	late Toarcian	Polymorphum Zone	limestone	15 m	Iznalloz, Spain	27	N0-30	2
175	late Toarcian	Rosso Ammonitico	limestone		ValdMRBia, Italy	28	N0-30	2
175	Toarcian	Rosso Ammonitico	limestone	27 m	Ticino, Switzerland	29	N30-60	2

182.7-170 .3	Toarcian-Aalenian	Rosso Ammonitico	limestone	35 m	Ionian Basin, Greece	30, 31	N0-30	2
182.7-170 .3	Toarcian-Aalenian	Rosso Ammonitico	limestone	30 m	Ionian Basin, Greece	32	N0-30	2
~189	Pliensbachian	Lower Senkoy Formation	limestone, mudstone	~18 m	Senkoy, Turkey	33	N30-60	2
~189	Pliensbachian	Lower Senkoy Formation	limestone	~22 m	Gokdere, Turkey	33	N30-60	2
189	early Pliensbachian	Ammonitico Rosso	limestone		Anatolia, Turkey	34	N30-60	2
195	Sinemurian	Ammonitico Rosso	limestone	~16 m	Montecatini, Italy	35	N0-30	2
195	Sinemurian	Ammonitico Rosso	limestone, mudstone	~26 m	La Spezia, Italy	35	N0-30	2
199-185	late Hettangian-early Pliensbachian	Ammonitico Rosso	limestone		Transdanubian Central Range, Hungary	36	N30-60	2
199-185	Hettangian-Pliensbachian	Pisznice Limestone Formation	limestone	~20 m	Transdanubian Central Range, Hungary	37	N30-60	2
199-185	Hettangian-Pliensbachian	Lower Senkoy Formation	limestone	~20 m	Canayurdu, Turkey	33	N30-60	2
199-185	Hettangian-Pliensbachian	Lower Senkoy Formation	limestone	~29 m	Tersun, Turkey	33	N30-60	2
199-185	Hettangian-Pliensbachian	Lower Senkoy Formation	limestone	~60 m	Kirikli, Turkey	33	N30-60	2
199-185	Hettangian-Pliensbachian	Lower Senkoy Formation	limestone	~50 m	Duragiza, Turkey	33	N30-60	2
199	Hettangian	Ammonitico Rosso	limestone		Transdanubian Central Range, Hungary	38	N30-60	2
~248	Olenekian	Nanlinghu Formation	limestone, mudstone	20 m	Chaohu, South China	This study	N0-30	2
~248	Spathian	Jialingjiang Formation	limestone	16 m	Wulong, South China	This study	S0-30	2
~248	Spathian	Luolou Formation	limestone	15 m	Lalaichao, South China	This study	S0-30	2
~248	Spathian	Luolou Formation	limestone	15 m	Guandao, South China	This study	S0-30	2
~248	Spathian	Luolou Formation	limestone	15 m	Mingtang, South China	This study	S0-30	2
~248	Spathian	Luolou Formation	limestone	2 m	Bianyang, South China	This study	S0-30	2
~248	Spathian	Luolou Formation	limestone	2 m	Qingyan, South China	This study	S0-30	2
~248	Spathian	Jialingjiang Formation	limestone	2 m	Zunyi, South China	This study	S0-30	2
~248	Spathian	Kangshare Formation	limestone, shale	10 m	Tulong, Tibet	This study	S30-60	2
~248	Spathian	Kangshare	limestone,	9 m	Yalai, Tibet	This study	S30-60	2

		Formation	shale					
~248	Spathian	*Neospathodus homeri* Zone	claystone	2 m	Aichi Prefecture, Japan	39	N0-30	2
~248	Spathian	Moenkopi Formation	limestone, shale	< 1 m	California, USA	This study	N0-30	2
~370	Famenian	Lower Three Forks Formation	limestone	33 m	Montana and Wyoming, USA	40	S0-30	2
~370	Famenian	Upper Shetianqiao Formation	limestone	4.3 m	Baqi, South China	41	N0-30	2
~370	Famenian	Wuzhishan Formation	limestone	66 m	Baisha, South China	This study	N0-30	2
~370	Famenian	Wuzhishan Formation	limestone	12 m	Lengshuihe, South China	This study	N0-30	2
~370	Famenian	Nullara Limestone	limestone	5.5 m	Canning basin, Western Australia	42	S0-30	2
~370	Famenian	Marginifer Zone, Trachytera Zone	limestone	1.5 m	Enkenberg, Germany	43	N0-30	2
~370	Frasnian-Famenian		limestone	2 m	Vogelsberg, Germany	44	N0-30	2
~370	Famenian	Cheiloceras beds	limestone	2 m	Casey Falls, Western Australia	45	S0-30	2
~370	Famenian	Cheiloceras beds	limestone	2 m	McWhae Ridge, Western Australia	45	S0-30	2
~370	Famenian	Sulcifer Formation	limestone	20-120 m	Central Kazakhstan	46	N30-60	2
372-370	Frasnian-Famenian	Coumiac carbonate	limestone	10 m	Coumiac, France	47	S0-30	2
494	Jiangshangian	Wilberns Limestone	limestone	4 m	White Creek, Texas, USA	48	S0-30	2
494	Jiangshangian	Wilberns Limestone	limestone	33 m	Lion Mountain, Texas, USA	48	S0-30	2
~506	Stage 5	Olenek Formation	limestone		North-Central Siberia	49	S0-30	2
~509	Stage 5	Mantou Formation	mudstone, shale		Shandong, North China	50	N30-60	2
~509	Stage 5	Koruk Formation	limestone, mudstone	17 m	Southeast Turkey	51	S30-60	2
~509	Stage 5	upper Cal Tepe Formation	limestone	47 m	Southwestern Turkey	52	S30-60	2
~509	Stage 5	upper Lancara Formation	limestone		Cantabrian, Spain	53	S30-60	2
~509	Stage 5	Lancara Formation	limestone		Spain	54	S30-60	2
~509	Stage 5	upper Cal Tepe Formation	limestone		Southwestern Turkey	54	S30-60	2
~510	Stage 4	middle Montejinni Limestone	limestone, mudstone		Northern Territory, Australia	55	S0-30	2

~516	Stage 3	Botomian Wilkawillina Limestone	limestone		Flinders Ranges, South Australia	56	S0-30	2
~520	Stage 3	Salaany Gol Formation	limestone	15 m	Zavkhan Basin, Mongolia	57	S0-30	2
~520	Stage 3	Little Hollow Formation	limestone		Nova Scotia, Canada	58	N0-30	2
520	Stage 3	early Atdabanian	limestone	12 m	Ulakhan-Kyyry-Taas, Siberian	59	S0-30	2
524-521	Stage 2	Tommotian	limestone	22 m	Zhurinskii Mys, Siberian	59	S0-30	2
524-521	Stage 2	Tommotian	limestone	75 m	Dvortsy, Siberian	59	S0-30	2
524	Stage 2	early Tommotian	limestone		Sukharikha River, Siberian	59	S0-30	2
~526	Stage 2	Member 4 of Chapel Island Formation	limestone, shale		Dantzic Cove, Newfoundland, Canada	60, 61	S0-30	2
~526	Stage 2	Member 4 of Chapel Island Formation	mudstone	~40 m	Dantzic Cove, Newfoundland, Canada	62	S0-30	2
~526	Stage 2	Member 4 of Chapel Island Formation	mudstone	~50 m	Fortune North, Newfoundland, Canada	62	S0-30	2
~526	Stage 2	Member 4 of Chapel Island Formation	mudstone	~50 m	Fortune North, Newfoundland, Canada	63	S0-30	2
571 ± 8	middle Ediacaran	Tikhfist Formation	limestone	>1 m	Anti-Atlas, Morocco	64	S30-60	65
580-550	middle Ediacaran	Johnnie Formation	limestone	< 1 m	northern Spring Mountains, USA	66	S30-60	65
580-550	middle Ediacaran	Johnnie Formation	limestone	< 1 m	Resting Spring Range, USA	66	S30-60	65
580-550	middle Ediacaran	Johnnie Formation	limestone	15 m	southern Nopah Range, USA	66, 67	S30-60	65
580-550	middle Ediacaran	Johnnie Formation	limestone	10 m	Johnson Canyon, USA	66	S30-60	65
580-550	middle Ediacaran	Johnnie Formation	limestone	4 m	Old Dad Mountains, USA	66	S30-60	65
580-550	middle Ediacaran	Johnnie Formation	sandy limestone	40 m	northern Mesquite Mountains, USA	This Study	S30-60	65
580-550	middle Ediacaran	Krol B (or Jarashi Formation)	shale, dolostone	≤40 m	Lesser Himalaya, India	68	S0-30	65
600-560	middle Ediacaran	Chenchinskaya Formation	limestone	30-50 m	Zhuya, Siberian	69	S0-30	65
600-560	middle Ediacaran	Alyanchskaya Formation	limestone	40 m	Bol'shoy Patom, Siberian	69	S0-30	65
620-550	middle Ediacaran	Lubudi Formation	limestone	3-10 m	Lubudi and Lukafu, Congo	70	S0-30	65
632-551	middle Ediacaran	Doushantuo	dolomite,	8 m	Yichang, South China	This Study	N30-60	65

		Formation	limestone					
790-660	Tonian	Braemar Ironstone	ironstone		South Australia	71	N0-30	65
767 ± 15	Tonian	Erzin BIF	ironstone	9 m	Erzin basin, Tuva	72	N30-60	65
>717 ± 3	Tonian	Pocatello Formation	ironstone	25 m	Idaho, North America	73	S0-30	65
730	Tonian	Fulu Formation	ironstone	3.4 m	Sanjiang, South China	This study	N30-60	65
730	Tonian	Fulu Formation	ironstone	25 m	Zhongjiajiang, South China	74	N30-60	65
730	Tonian	Fulu Formation	ironstone	50 m	Lanyang, South China	74	N30-60	65
730	Tonian	Fulu Formation	ironstone	30 m	Jinzhai, South China	74	N30-60	65
730	Tonian	Fulu Formation	ironstone	10 m	Changanbao, South China	74	N30-60	65
730	Tonian	Fulu Formation	ironstone	35 m	Longjia, South China	74	N30-60	65
730	Tonian	Fulu Formation	ironstone	15 m	Lijiapo, South China	74	N30-60	65
730	Tonian	Fulu Formation	ironstone	50 m	Yihuang, South China	This study	N30-60	65
730	Tonian	Rapitian Group	ironstone	150 m	Snake River, Canada	75	N0-30	65
730	Tonian	Rapitian Group	ironstone	30 m	Tatonduk River, Canada	75	N0-30	65
730	Tonian	Rapitian Group	ironstone	25 m	Backbone Range, Canada	75	N0-30	65
730	Tonian	Rapitian Group	ironstone	20 m	Thundercloud Range, Canada	75	N0-30	65
730	Tonian	Santa Cruz Formation	ironstone		Urucum, Brazil	76	S30-60	65
730	Tonian	Banda Alta Formation	ironstone	~300 m	Urucum, Brazil	77	S30-60	65
731 ± 4	Tonian	Wadi Kareim BIF	ironstone	6 m	Eastern Desert, Egypt	78	S0-30	65
734 ± 7	Tonian	Wadi El Dabbah BIF	ironstone	50 m	Eastern Desert, Egypt	78	S0-30	65
~750	Tonian	Chuos Formation	ironstone	50 m	Owambo Basin, Namibia	79	S0-30	65
1738.5 ± 0.5	Statherian	Cleopatra Rhyolite	ironstone	15 m	central Arizona, USA	80	N30-60	81
1874 ± 9	Orosirian	Vulcan Iron Formation, Menominee Group	ironstone		Great Lake, North America	82, 83	N30-60	81
1877.8 ± 1.3	Orosirian	Sokoman Iron Formation	ironstone		Labrador Trough, North America	84	N30-60	81
1878 ± 2	Orosirian	Gunflint Iron Formation, Animikie Group	ironstone	120 m	Lake Superior, North America	85	N30-60	81
1880	Orosirian	Sokoman Iron Formation	ironstone	150 m	Labrador Trough, North America	86	N30-60	81
1891± 8	Orosirian	Frere Formation	ironstone	600 m	Earahedy Basin, Western Australia	87, 88	N30-60	81
1910 ± 10	Orosirian	Fence River Formation, Menominee Group	ironstone		Lake Superior, North America	85	N30-60	81

1914 ± 120	Orosirian	Morar Formation, Gwalior Group	ironstone	600 m	Gwalior basin, India	89	S0-30	90
2460 ± 5	Siderian	Griquatown BIF, Kuruman BIF, Ghaap Group	ironstone	250 m	Transvaal basin, South Afirca	91, 92, 93, 94	S0-30	81
~2460	Siderian	Cauê Formation	ironstone	350 m	Minas Gerais, Brazil	95, 96	S0-30	81
2480 ± 6	Siderian	Penge BIF, Chuniespoort Group	ironstone		Transvaal basin, South Afirca	93	S0-30	81
2481 ± 4	Siderian	Brockman Iron Formation	ironstone	600 m	Western Australia	97, 98, 99, 100	S30-60	81
2533 ± 11	Neoarchean	Anshan Group	ironstone	140 m	Gongchangling, North China	101	S60-90	102
2534 ± 8	Neoarchean	Luanxian Group	ironstone	161 m	Miyun, North China	103, 104, 105	S60-90	102
2545 ± 7	Neoarchean	Xinghe Group	ironstone		Guyang, North China	105	S60-90	102
2549 ± 7	Neoarchean	Proto BIF, Nauga Formation	ironstone	30 m	Prieska, South Afirca	106	S60-90	102
~2550	Neoarchean	Baizhiyan Formation	ironstone	38 m	Lvliang, North China	This study	S60-90	102
~2550	Neoarchean	Yuanjiacun Formation	ironstone	500 m	Wutai, North China	This study	S60-90	102
~2550	Neoarchean	Rio Das Velhas Supergroup	ironstone	200 m	Minas Gerais, Brazil	96	S0-30	81
2554 ± 10	Neoarchean	Kolar Group	ironstone		Kolar, India	107, 108	S60-90	109
2555 ± 7	Neoarchean	Jianping Group	ironstone		Jianping, North China	110	S60-90	102
2629 ± 4	Neoarchean	Wittenoom Formation	ironstone	150 m	Western Australia	97, 98, 99, 100, 111, 112	S30-60?	113
2677 ± 2	Neoarchean	Chitradurga Group	ironstone		Chitradurga, India	108, 114	S30-60?	
2691 ± 9	Neoarchean	Koolyanobbing Greenstone Belt	ironstone	200 m	Western Australia	115, 116, 117, 118	S30-60?	113
2718 ± 6	Neoarchean	Bababudan Group	ironstone		Karnataka, India	114, 119	S30-60?	
2731 ± 2	Neoarchean	North Spirit Lake greenstone belt	ironstone		northwestern Ontario, Canada	120	S0-30?	
2747 ± 1	Neoarchean	Carajás BIF	ironstone	250 m	Carajás, Brazil	121	S0-30?	
2847 ± 4	Mesoarchean	Itilliarsuup Qaqqaa BIF	ironstone	400 m	Disko Bay, West Greenland	122	S30-60?	
2914 ± 8	Mesoarchean	West Rand Group	ironstone	minor	Witwatersrand basin, South Afirca	123	S60-90?	113
2990 ± 7	Mesoarchean	Mosquito Creek Formation	ironstone		Western Australia	124	S30-60?	113
3014 ± 13	Mesoarchean	Western Gneiss Terrain	ironstone		Western Australia	125	S30-60?	113
3112 ± 6	Mesoarchean	Cleaverville	ironstone		Western Australia	124, 126	S30-60?	113

		Formation						
3235 ± 3	Paleoarchean	Nimmingarra Iron Formation	ironstone	50 m	Western Australia	100, 127	S30-60?	113
3243 ± 4	Paleoarchean	Jaspilite BIF	ironstone	40 m	Barberton, South Afirca	100, 128	S60-90?	113
3298 ± 7	Paleoarchean	Sardur Group	ironstone		Karnataka, India	114, 129	S30-60?	
3506.8 ± 2.3	Paleoarchean	Iron Ore Group	ironstone	120 m	Singhbhum, India	130	S30-60?	
3689 ± 5	Eoarchean	Isua Supracrustal Belt	ironstone	5 m	Nuuk, West Greenland	131, 132	S30-60?	
3802 ± 12	Eoarchean	Nuvvuagittuq Supracrustal Belt	ironstone	35 m	Quebec, Canada	133	S30-60?	
3850	Eoarchean	Itsaq Gneiss Complex	ironstone	20 m	Akilia, West Greenland	134	S30-60?	

Supplementary Table 3｜Carbon isotope data used in Figure 2c.

Age (Ma)	Era	Period	Stage	Strata unite	Section	Region	References
84	Mesozoic	Cretaceous	Campanian	Chuangde Formation	Chuangde	Tibet	This study
94	Mesozoic	Cretaceous	Turonian	Red beds	Buchberg	Austria	10
94	Mesozoic	Cretaceous	Turonian	Scaglia Rossa Formation	Gubbio	Italy	167
94	Mesozoic	Cretaceous	Turonian	Scaglia Rossa Formation	Gubbio	Italy	168
94	Mesozoic	Cretaceous	Turonian	Red beds	Buchberg	Austria	169
94	Mesozoic	Cretaceous	Turonian	Red beds	Eglish Chalk	UK	170
113	Mesozoic	Cretaceous	Aptian	Scisti a Fucoidi Formation	Gorge a Cerbara	Italy	171
113	Mesozoic	Cretaceous	Aptian	Red beds	Yenicesihlar	Turkey	172
152	Mesozoic	Jurassic	Tithonian	Rosso Ammonitico	Monte Inici	Sicily	18
157	Mesozoic	Jurassic	Oxfordian-Kimmeridgian	Rosso Ammonitico	Monte Inici	Sicily	18
168	Mesozoic	Jurassic	Bajocian-Bathonian	Rosso Ammonitico	Puerto Escano	Spain	173
199	Mesozoic	Jurassic	Hettangian-Sinemurian	Ammonitico Rooso	Montecatini	Italy	35
199	Mesozoic	Jurassic	Hettangian-Sinemurian	Moltrasio Formation and Sedrina limestone	Pozzo Glaciale	Italy	174
248	Mesozoic	Triassic	Spathian	Luolou Formation	Mingtang	South China	This study
370	Paleozoic	Devonian	Famennian	Wuzhishan Formation	Baisha	South China	This study
509	Paleozoic	Cambrian	Stage 5	La Tanque Formation	Ferrals-les-Montagnes	South France	175
520	Paleozoic	Canbrian	Tommotian	Pestrotsvet Formation	Dvortsy	Siberian	176
580	Neoproterozoic	Ediacarian		Doushantuo Formation	Shijiahe	South China	This study
580	Neoproterozoic	Ediacarian		Johnnie Formation	North Mesquite Mountains	USA	This study
730	Neoproterozoic	Tonian		Fulu Formation	Sangyuan	South China	This study
730	Neoproterozoic	Tonian		Santa Cruz Formation	Urucum District	Brazil	76
1900	Paleoproterozoic	Orosirian		Gunflint Iron Formation	Thunder Bay	Canada	177
2460	Paleoproterozoic	Siderian		Kuruman Iron Formation	Adelaide Pomfret	South Africa	178
2500	Neoarchean			Baizhiyan Formation	Kangjiagou	North China	This study
2500	Neoarchean			Brockman Iron Formation	Hamersley Range	Western Australia	111
2600	Neoarchean			Mount Sylvia Iron Formation	Hamersley Range	Western Australia	179
3000	Mesoarchean			Swaziland Sequence	Barberton Mountain Land	South Africa	180
3800	Eoarchean				Isua Supracrustal Belt	Southwest Greenland	181

Supplementary References

1. Zeng X. The temporal and spatial evolution of CORBs. Chengdu University of Technology, Chengdu, 2006.

2. Scotese CR. *Atlas of earth history, Volume 1, Paleogeography*. PALEOMAP Project: Arlington, 2001.

3. Wang CS, Hu XM, Huang Y, Scott RW, Wagreich M. Overview of Cretaceous Oceanic Red Beds (CORBs): a window on global oceanic and climate change. In: Hu XM, Wang CS, Scott RW, Wagreich M, Jansa L (eds). *Cretaceous Oceanic Red Beds: Stratigraphy, Composition, Origins and Paleoceanographic and Paleoclimatic Significance: SEPM Special Publication*, vol. 91, 2009, pp 13-33.

4. Wan X, Lamolda MA, Si J, Li G. Foraminiferal stratigraphy of Late Cretaceous red beds in southern Tibet. *Cretaceous Research* 2005, **26**(1)**:** 43-48.

5. Chen X. Ages, lithofacies and depositional environments of Cretaceous Oceanic Red Beds: review and a case study in Gyangze Basin, southern Tibet. China University of Geosciences, Beijing, 2009.

6. Li G, Jiang G, Wan X. The age of the Chuangde Formation in Kangmar, southern Tibet of China: Implications for the origin of Cretaceous oceanic red beds (CORBs) in the northern Tethyan Himalaya. *Sedimentary Geology* 2011, **235**(1–2)**:** 111-121.

7. Yilmaz İÖ. Cretaceous pelagic red beds and black shales (Aptian-Santonian), NW Turkey: Global oceanic anoxic and oxic events. *Turkish Journal of Earth Sciences* 2008, **17**(2)**:** 263-296.

8. Hu X, Jansa L, Wang C, Sarti M, Bak K, Wagreich M*, et al.* Upper Cretaceous oceanic red beds (CORBs) in the Tethys: occurrences, lithofacies, age, and environments. *Cretaceous Research* 2005, **26**(1)**:** 3-20.

9. Hu X, Jansa L, Sarti M. Mid-Cretaceous oceanic red beds in the Umbria–Marche Basin, central Italy: constraints on paleoceanography and paleoclimate. *Palaeogeography, Palaeoclimatology, Palaeoecology* 2006, **233**(3–4)**:** 163-186.

10. Neuhuber S, Wagreich M, Wendler I, Spötl C. Turonian Oceanic Red Beds in the Eastern Alps: concepts for palaeoceanographic changes in the Mediterranean Tethys. *Palaeogeography, Palaeoclimatology, Palaeoecology* 2007, **251**(2)**:** 222-238.

11. Melinte-Dobrinescu MC, Roban R-D. Cretaceous anoxic–oxic changes in the Moldavids (Carpathians, Romania). *Sedimentary Geology* 2011, **235**(1–2)**:** 79-90.

12. Li X, Hu X, Cai Y, Han Z. Quantitative analysis of iron oxide concentrations within Aptian–Albian cyclic oceanic red beds in ODP Hole 1049C, North Atlantic. *Sedimentary Geology* 2011, **235**(1–2)**:**

91-99.

13. Lukeneder A. The Biancone and Rosso Ammonitico facies of the northern Trento Plateau (Dolomites, Southern Alps, Italy). *Annelan des Naturhistorischen Museum Wien A* 2011, **113:** 9-33.

14. Mamet B, Préat A. Jurassic microfacies, Rosso Ammonitico limestone, Subbetic Cordillera, Spain. *Revista Española de Micropaleontología* 2006, **38:** 219.

15. Coimbra R, Immenhauser A, Olóriz F. Matrix micrite $\delta^{13}C$ and $\delta^{18}O$ reveals synsedimentary marine lithification in Upper Jurassic Ammonitico Rosso limestones (Betic Cordillera, SE Spain). *Sedimentary Geology* 2009, **219**(1)**:** 332-348.

16. Seyed-Emami K, Schairer G, Raoufian A, Shafeizad M. Middle and Late Jurassic ammonites from the Dalichai Formation west of Shahrud (East Alborz, North Iran). *Neues Jahrbuch für Geologie und Paläontologie-Abhandlungen* 2013, **267**(1)**:** 43-66.

17. Zavala C. Tracking sea bed topography in the Jurassic. The Lotena Group in the Sierra de la Vaca Muerta (Neuquén Basin, Argentina). *Geologica Acta* 2005, **3**(2)**:** 107-118.

18. Cecca F, Savary B, Bartolini A, Remane J, Cordey F. The Middle Jurassic-Lower Cretaceous Rosso Ammonitico succession of Monte Inici (Trapanese Domain, western Sicily); sedimentology, biostratigraphy and isotope stratigraphy. *Bulletin de la Societe Geologique de France* 2001, **172**(5)**:** 647-659.

19. Mamet B, Préat A. On the bacterial and fungal origin of the Ammonitico Rosso red pigmentation (Jurassic, Verone area, northern Italy). *Revue de Micropaleontologie* 2003, **46**(1)**:** 35-46.

20. Préat A, Morano S, Loreau J-P, Durlet C, Mamet B. Petrography and biosedimentology of the Rosso Ammonitico Veronese (middle-upper Jurassic, north-eastern Italy). *Facies* 2006, **52**(2)**:** 265-278.

21. Martire L. Stratigraphy, facies and synsedimentary tectonics in the Jurassic Rosso Ammonitico Veronese (Altopiano di Asiago, NE Italy). *Facies* 1996, **35**(1)**:** 209-236.

22. Šmuc A, Rožič B. The Jurassic Prehodavci Formation of the Julian Alps: easternmost outcrops of Rosso Ammonitico in the Southern Alps (NW Slovenia). *Swiss Journal of Geosciences* 2010, **103**(2)**:** 241-255.

23. Minoura K, Nakaya SHU, Takemura A. Origin of manganese carbonates in Jurassic red shale, central Japan. *Sedimentology* 1991, **38**(1)**:** 137-152.

24. Preat A, Mamet B, Di Stefano P, Martire L, Kolo K. Microbially-induced Fe and Mn oxides in condensed pelagic sediments (Middle-Upper Jurassic, Western Sicily). *Sedimentary Geology* 2011, **237**(3)**:** 179-188.

25. Di Stefano P, Mindszenty A. Fe–Mn-encrusted “Kamenitza" and associated features in the Jurassic of Monte Kumeta (Sicily): subaerial and/or submarine dissolution? *Sedimentary Geology* 2000, **132**(1–2)**:** 37-68.

26. Varol B, Gökten E. The facies properties and depositional environments of nodular limestones and red marly limestones (Ammonitico Rosso) in the Ankara Jurassic sequence, central Turkey. *Terra Nova* 1994, **6**(1)**:** 64-71.

27. Galbrun B, Baudin F, Fourcade E, Rivas P. Magnetostratigraphy of the Toarcian ammonitico rosso limestone at Iznalloz, Spain. *Geophysical Research Letters* 1990, **17**(13)**:** 2441-2444.

28. Sabatino N, Neri R, Bellanca A, Jenkyns HC, Baudin F, Parisi G*, et al.* Carbon‐isotope records of the Early Jurassic (Toarcian) oceanic anoxic event from the Valdorbia (Umbria-Marche Apennines) and Monte Mangart (Julian Alps) sections: Palaeoceanographic and stratigraphic implications. *Sedimentology* 2009, **56**(5)**:** 1307-1328.

29. Horner F, Heller F. Lower Jurassic magnetostratigraphy at the Breggia Gorge (Ticino, Switzerland) and Alpe Turati (Como, Italy). *Geophysical Journal International* 1983, **73**(3)**:** 705-718.

30. Karakitsios V. Evolution and Petroleum Potential of the Ionian Basin (Northwest Greece). International Conference & Exhibition, AAPG; 2003; 2003. p. 21-24.

31. Karakitsios V. The influence of preexisting structure and halokinesis on organic matter preservation and thrust system evolution in the Ionian Basin, Northwest Greece. *AAPG bulletin* 1995, **79**(7)**:** 960-980.

32. Rigakis N, Karakitsios V. The source rock horizons of the Ionian Basin (NW Greece). *Marine and Petroleum Geology* 1998, **15**(7)**:** 593-617.

33. Kandemir R, Yılmaz C. Lithostratigraphy, facies, and deposition environment of the lower Jurassic Ammonitico Rosso type sediments (ARTS) in the Gümüşhane area, NE Turkey: implications for the opening of the northern branch of the Neo-Tethys Ocean. *Journal of Asian Earth Sciences* 2009, **34**(4)**:** 586-598.

34. Cope J. Ammonite faunas of the Ammonitico Rosso of the Pontide Mountains, northern Anatolia. *Geologica Romana* 1991, **27:** 303-325.

35. Van de Schootbrugge B, Payne J, Tomasovych A, Pross J, Fiebig J, Benbrahim M*, et al.* Carbon cycle perturbation and stabilization in the wake of the Triassic‐Jurassic boundary mass‐extinction event. *Geochemistry, Geophysics, Geosystems* 2008, **9**(4)**:** 1-16.

36. Gorog Á. Early Jurassic planktonic foraminifera from Hungary. *Micropaleontology* 1994, **40**(3)**:** 255-260.

37. Haas J, Hámor G. Geological garden in the neighborhood of Budapest, Hungary. *Episodes* 2001, **24**(4)**:**

257-261.

38. Blau J, Haas Á. Lower Liassic involutinids (foraminifera) from the Transdanubian Central Range, Hungary. *Paläontologische Zeitschrift* 1991, **65**(1-2)**:** 7-23.

39. Takahashi S, Oba M, Kaiho K, Yamakita S, Sakata S. Panthalassic oceanic anoxia at the end of the Early Triassic: A cause of delay in the recovery of life after the end-Permian mass extinction. *Palaeogeography Palaeoclimatology Palaeoecology* 2009, **274**(3-4)**:** 185-195.

40. Sandberg CA. Nomenclature and correlation of lithologic subdivisions of the Jefferson and Three Forks Formations of southern Montana and northern Wyoming. *Geological Survery Bulletin* 1965, **1194:** 1-18.

41. Wang K, Bai S. Faunal changes and events near the Frasnian-Famennian boundary of South China. *Canadian Society of Petroleum Geologists* 1988, **14:** 71-78.

42. Hurley N, Van der Voo R. Paleomagnetism of Upper Devonian reefal limestones, Canning basin, western Australia. *Geological Society of America Bulletin* 1987, **98**(2)**:** 138-146.

43. Korn D, Ziegler W. The ammonoid and conodont zonation at Enkenberg (Famennian, Late Devonian; Rhenish Mountains). *Senckenbergiana lethaea* 2002, **82**(2)**:** 453-462.

44. Joachimski MM, Buggisch W. Anoxic events in the late Frasnian—Causes of the Frasnian-Famennian faunal crisis? *Geology* 1993, **21**(8)**:** 675-678.

45. Becker RT, House MR, Kirchgasser WT, Playford PE. Sedimentary and faunal changes across the Frasnian/Famennian boundary in the Canning Basin of Western Australia. *Historical Biology* 1991, **5**(2-4)**:** 183-196.

46. Veimarn A, Vorontzova T, Martynova M. Stratigraphy, paleogeography and iron-manganese ores of the Famennian of central Kazakhstan. *Proceedings of the 2nd International Symposium on the Devonian System* 1988, **14:** 681-689.

47. Preat A, Mamet B, Devleeschouwer X. Sédimentologie du stratotype de la limite Frasnien-Famennien (Coumiac, Montagne Noire, France). *Bulletin de la Societe Geologique de France* 1998, **169**(3)**:** 331-342.

48. Wilson JL. The trilobite fauna of the Elvinia Zone in the basal Wilberns Limestone of Texas. *Journal of Paleontology* 1949**:** 25-44.

49. Lazarenko NP. Middle and Upper Cambrian Strata of North-Central Siberia. *Regional Arctic Geology of the USSR* 1973**:** 291-295.

50. Chough SK, Lee HS, Woo J, Chen J, Choi DK, Lee S-b*, et al.* Cambrian stratigraphy of the North

China Platform: revisiting principal sections in Shandong Province, China. *Geosciences Journal* 2010, **14**(3)**:** 235-268.

51. Dean WT. Cambrian Stratigraphy and Trilobites of the Samur Da¤ Area, South of Hakkâri, Southeastern Turkey. *Turkish Journal of Earth Sciences (Turkish J Earth Sci)* 2006, **15:** 225-257.

52. Dean WT. Trilobites from the Çal Tepe Formation (Cambrian), near Seydişehir, Central Taurides, southwestern Turkey. *Turkish Journal of Earth Sciences* 2005, **14**(1)**:** 1-71.

53. Sdzuy K, Liñán E, Gozalo R. The Leonian Stage (early Middle Cambrian): a unit for Cambrian correlation in the Mediterranean subprovince. *Geological Magazine* 1999, **136**(01)**:** 39-48.

54. Fernández-Remolar DC. Latest Neoproterozoic to Middle Cambrian body fossil record in Spain (exclusive of trilobites and archaeocyaths) and their stratigraphic significance. *GFF* 2001, **123**(2)**:** 73-80.

55. Luck G. The palaeomagnetism of some Cambrian and Ordovician sediments from the Northern Territory, Australia. *Geophysical Journal International* 1970, **20**(1)**:** 31-39.

56. James NP, Gravestock DI. Lower Cambrian shelf and shelf margin buildups, Flinders Ranges, South Australia1. *Sedimentology* 1990, **37**(3)**:** 455-480.

57. Kruse PD, Gandin A, Debrenne F, Wood R. Early Cambrian bioconstructions in the Zavkhan Basin of western Mongolia. *Geological Magazine* 1996, **133**(04)**:** 429-444.

58. Landing E, Nowlan GS, Fletcher TP. A microfauna associated with Early Cambrian trilobites of the Callavia Zone, norther Antigonish Highlands, Nova Scotia. *Canadian Journal of Earth Sciences* 1980, **17**(3)**:** 400-418.

59. Rozanov AY, Khomentovsky V, Shabanov YY, Karlova G, Varlamov A, Luchinina V*, et al.* To the problem of stage subdivision of the Lower Cambrian. *Stratigraphy and geological correlation* 2008, **16**(1)**:** 1-19.

60. Landing E. Lower Cambrian of eastern Massachusetts: stratigraphy and small shelly fossils. *Journal of Paleontology* 1988**:** 661-695.

61. McIlroy D, Szaniawski H. A lower Cambrian protoconodont apparatus from the Placentian of southeastern Newfoundland. *Lethaia* 2000, **33**(2)**:** 95-102.

62. Myrow PM. Mixed siliciclastic-carbonate deposition in an Early Cambrian oxygen-stratified basin, Chapel Island Formation, southeastern Newfoundland. *Journal of Sedimentary Research* 1992, **62**(3).

63. Strauss H, Bengtson S, Myrow PM, Vidal G. Stable isotope geochemistry and palynology of the late Precambrian to Early Cambrian sequence in Newfoundland. *Canadian Journal of Earth Sciences* 1992,

29(8)**:** 1662-1673.

64. Álvaro JJ, Ezzouhairi H, Ayad NA, Charif A, Solá R, Ribeiro ML. Alkaline lake systems with stromatolitic shorelines in the Ediacaran volcanosedimentary Ouarzazate Supergroup, Anti-Atlas, Morocco. *Precambrian research* 2010, **179**(1–4)**:** 22-36.

65. Li Z-X, Bogdanova S, Collins AS, Davidson A, De Waele B, Ernst R*, et al.* Assembly, configuration, and break-up history of Rodinia: a synthesis. *Precambrian research* 2008, **160**(1)**:** 179-210.

66. Bergmann KD, Zentmyer RA, Fischer WW. The stratigraphic expression of a large negative carbon isotope excursion from the Ediacaran Johnnie Formation, Death Valley. *Precambrian research* 2011, **188**(1–4)**:** 45-56.

67. Pruss SB, Corsetti FA, Fischer WW. Seafloor-precipitated carbonate fans in the Neoproterozoic Rainstorm Member, Johnnie Formation, Death Valley Region, USA. *Sedimentary Geology* 2008, **207**(1–4)**:** 34-40.

68. Jiang G, Christie-Blick N, Kaufman AJ, Banerjee DM, Rai V. Sequence Stratigraphy of the Neoproterozoic Infra Krol Formation and Krol Group, Lesser Himalaya, India. *Journal of Sedimentary Research* 2002, **72**(4)**:** 524-542.

69. Melezhik VA, Pokrovsky BG, Fallick AE, Kuznetsov AB, Bujakaite MI. Constraints on $^{87}Sr/^{86}Sr$ of Late Ediacaran seawater: insight from Siberian high-Sr limestones. *Journal of the Geological Society* 2009, **166**(1)**:** 183-191.

70. Batumike MJ, Cailteux JLH, Kampunzu AB. Lithostratigraphy, basin development, base metal deposits, and regional correlations of the Neoproterozoic Nguba and Kundelungu rock successions, central African Copperbelt. *Gondwana Research* 2007, **11**(3)**:** 432-447.

71. Preiss WV, Gostin VA, McKirdy DM, Ashley PM, Williams GE, Schmidt PW. Chapter 69 The glacial succession of Sturtian age in South Australia: the Yudnamutana Subgroup. *Geological Society, London, Memoirs* 2011, **36**(1)**:** 701-712.

72. Ilyin A. Neoproterozoic banded iron formations. *Lithology and Mineral Resources* 2009, **44**(1)**:** 78-86.

73. Fanning CM, Link PK. U-Pb SHRIMP ages of Neoproterozoic (Sturtian) glaciogenic Pocatello Formation, southeastern Idaho. *Geology* 2004, **32**(10)**:** 881-884.

74. Lin S, Xiao J, Lu D, Liu A, Mou S, Cheng R*, et al.* Re-division about Fulu Formation and Fulu interglacial epoch in Hunan-Guizhou-Guangxi border area in South China. *Geological Bulletin of China* 2010, **29**(2-3)**:** 195-204.

75. Klein C, Beukes NJ. Sedimentology and geochemistry of the glaciogenic late Proterozoic Rapitan iron-formation in Canada. *Economic Geology* 1993, **88**(3)**:** 542-565.

76. Klein C, Ladeira EA. Geochemistry and mineralogy of Neoproterozoic banded iron-formations and some selected, siliceous manganese formations from the Urucum District, Mato Grosso do Sul, Brazil. *Economic Geology* 2004, **99**(6)**:** 1233-1244.

77. Freitas BT, Warren LV, Boggiani PC, De Almeida RP, Piacentini T. Tectono-sedimentary evolution of the Neoproterozoic BIF-bearing Jacadigo Group, SW-Brazil. *Sedimentary Geology* 2011, **238**(1–2)**:** 48-70.

78. Ali KA, Stern RJ, Manton WI, Kimura J-I, Khamees HA. Geochemistry, Nd isotopes and U–Pb SHRIMP zircon dating of Neoproterozoic volcanic rocks from the Central Eastern Desert of Egypt: New insights into the ∼750 Ma crust-forming event. *Precambrian research* 2009, **171**(1–4)**:** 1-22.

79. Le Heron DP, Busfield ME, Le Ber E, Kamona AF. Neoproterozoic ironstones in northern Namibia: Biogenic precipitation and Cryogenian glaciation. *Palaeogeography, Palaeoclimatology, Palaeoecology* 2013, **369:** 48-57.

80. Slack JF, Grenne T, Bekker A, Rouxel OJ, Lindberg PA. Suboxic deep seawater in the late Paleoproterozoic: Evidence from hematitic chert and iron formation related to seafloor-hydrothermal sulfide deposits, central Arizona, USA. *Earth and Planetary Science Letters* 2007, **255**(1–2)**:** 243-256.

81. Pesonen L, Elming S-Å, Mertanen S, Pisarevsky S, D'Agrella-Filho M, Meert J*, et al.* Palaeomagnetic configuration of continents during the Proterozoic. *Tectonophysics* 2003, **375**(1)**:** 289-324.

82. Schneider D, Bickford M, Cannon W, Schulz K, Hamilton M. Age of volcanic rocks and syndepositional iron formations, Marquette Range Supergroup: implications for the tectonic setting of Paleoproterozoic iron formations of the Lake Superior region. *Canadian Journal of Earth Sciences* 2002, **39**(6)**:** 999-1012.

83. Bekker A, Karhu JA, Kaufman AJ. Carbon isotope record for the onset of the Lomagundi carbon isotope excursion in the Great Lakes area, North America. *Precambrian research* 2006, **148**(1–2)**:** 145-180.

84. Findlay JM, Parrish RR, Birkett TC, Watanabe DH. U-Pb ages from the Nimish Formation and Montagnais glomeroporphyritic gabbro of the central New Québec Orogen, Canada. *Canadian Journal of Earth Sciences* 1995, **32**(8)**:** 1208-1220.

85. Ojakangas RW, Morey GB, Southwick DL. Paleoproterozoic basin development and sedimentation in the Lake Superior region, North America. *Sedimentary Geology* 2001, **141–142:** 319-341.

86. Klein C. Some Precambrian banded iron-formations (BIFs) from around the world: Their age, geologic setting, mineralogy, metamorphism, geochemistry, and origins. *American Mineralogist* 2005, **90**(10)**:** 1473-1499.

87. Pirajno F, Hocking RM, Reddy SM, Jones AJ. A review of the geology and geodynamic evolution of the Palaeoproterozoic Earaheedy Basin, Western Australia. *Earth-Science Reviews* 2009, **94**(1)**:** 39-77.

88. Rasmussen B, Fletcher IR, Bekker A, Muhling JR, Gregory CJ, Thorne AM. Deposition of 1.88-billion-year-old iron formations as a consequence of rapid crustal growth. *Nature* 2012, **484**(7395)**:** 498-501.

89. Absar N, Raza M, Roy M, Naqvi SM, Roy AK. Composition and weathering conditions of Paleoproterozoic upper crust of Bundelkhand craton, Central India: Records from geochemistry of clastic sediments of 1.9 Ga Gwalior Group. *Precambrian research* 2009, **168**(3–4)**:** 313-329.

90. Pradhan VR, Meert JG, Pandit MK, Kamenov G, Gregory LC, Malone SJ. India's changing place in global Proterozoic reconstructions: A review of geochronologic constraints and paleomagnetic poles from the Dharwar, Bundelkhand and Marwar cratons. *Journal of Geodynamics* 2010, **50**(3–4)**:** 224-242.

91. Horstmann UE, Hälbich IW. Chemical composition of banded iron-formations of the Griqualand West Sequence, Northern Cape Province, South Africa, in comparison with other Precambrian iron formations. *Precambrian research* 1995, **72**(1–2)**:** 109-145.

92. Pickard AL. SHRIMP U–Pb zircon ages for the Palaeoproterozoic Kuruman Iron Formation, Northern Cape Province, South Africa: evidence for simultaneous BIF deposition on Kaapvaal and Pilbara Cratons. *Precambrian research* 2003, **125**(3–4)**:** 275-315.

93. Bekker A, Holland H, Wang P-L, Rumble D, Stein H, Hannah J*, et al.* Dating the rise of atmospheric oxygen. *Nature* 2004, **427**(6970)**:** 117-120.

94. Heimann A, Johnson CM, Beard BL, Valley JW, Roden EE, Spicuzza MJ*, et al.* Fe, C, and O isotope compositions of banded iron formation carbonates demonstrate a major role for dissimilatory iron reduction in ~2.5 Ga marine environments. *Earth and Planetary Science Letters* 2010, **294**(1–2)**:** 8-18.

95. Klein C, Ladeira E. Geochemistry and petrology of some Proterozoic banded iron-formations of the Quadrilátero Ferrífero, Minas Gerais, Brazil. *Economic Geology* 2000, **95**(2)**:** 405-427.

96. Spier CA, de Oliveira SMB, Sial AN, Rios FJ. Geochemistry and genesis of the banded iron formations of the Cauê Formation, Quadrilátero Ferrífero, Minas Gerais, Brazil. *Precambrian research* 2007, **152**(3–4)**:** 170-206.

97. Trendall A, Compston W, Nelson D, De Laeter J, Bennett V. SHRIMP zircon ages constraining the depositional chronology of the Hamersley Group, Western Australia. *Australian Journal of Earth Sciences* 2004, **51**(5)**:** 621-644.

98. Trendall AF. The significance of iron-formation in the Precambrian stratigraphic record. *Precambrian Sedimentary Environments: A Modern Approach to Ancient Depositional Systems, Special Publication*

2002, **33:** 33-66.

99. Pickard AL, Barley ME, Krapež B. Deep-marine depositional setting of banded iron formation: sedimentological evidence from interbedded clastic sedimentary rocks in the early Palaeoproterozoic Dales Gorge Member of Western Australia. *Sedimentary Geology* 2004, **170**(1–2)**:** 37-62.

100. Glikson A. Asteroid impact ejecta units overlain by iron-rich sediments in 3.5–2.4 Ga terrains, Pilbara and Kaapvaal cratons: Accidental or cause–effect relationships? *Earth and Planetary Science Letters* 2006, **246**(3–4)**:** 149-160.

101. Dai Y, Zhang L, Wang C, Liu L, Cui M, Zhu M*, et al.* Genetic type, formation age and tectonic setting of the Waitoushan banded iron formation, Benxi, Liaoning Province. *Acta Petrologica Sinica* 2012, **28**(11)**:** 3574-3594.

102. Zhao G, Sun M, Wilde SA. Correlations between the eastern block of the North China Craton and the South Indian block of the Indian shield: an Archaean to Palaeoproterozoic link. *Precambrian research* 2003, **122**(1)**:** 201-233.

103. Nutman AP, Wan Y, Du L, Friend CRL, Dong C, Xie H*, et al.* Multistage late Neoarchaean crustal evolution of the North China Craton, eastern Hebei. *Precambrian research* 2011, **189**(1–2)**:** 43-65.

104. Li H-m, Wang D-h, Li L-x, Chen J, Yang X-q, Liu M-j. Metallogeny of iron deposits and resource potential of major iron minerogenetic units in China. *Geology in China* 2012, **3:** 559-580.

105. Wan Y, Dong C, Xie H, Wang S, Song M, Xu Z*, et al.* Formation ages of early Precambrian BIFs in the North China Craton: SHRIMP zircon U–Pb dating. *Acta Geol Sin* 2012, **86**(12)**:** 1447-1478.

106. Kazmierczak J, Altermann W, Kremer B, Kempe S, Eriksson PG. Mass occurrence of benthic coccoid cyanobacteria and their role in the production of Neoarchean carbonates of South Africa. *Precambrian research* 2009, **173**(1–4)**:** 79-92.

107. Dey S. Evolution of Archaean crust in the Dharwar craton: The Nd isotope record. *Precambrian research* 2013, **227:** 227-246.

108. Jayananda M, Peucat JJ, Chardon D, Rao BK, Fanning CM, Corfu F. Neoarchean greenstone volcanism and continental growth, Dharwar craton, southern India: Constraints from SIMS U–Pb zircon geochronology and Nd isotopes. *Precambrian research* 2013, **227:** 55-76.

109. Halls H, Kumar A, Srinivasan R, Hamilton M. Paleomagnetism and U–Pb geochronology of easterly trending dykes in the Dharwar craton, India: feldspar clouding, radiating dyke swarms and the position of India at 2.37 Ga. *Precambrian research* 2007, **155**(1)**:** 47-68.

110. Liu S, Santosh M, Wang W, Bai X, Yang P. Zircon U–Pb chronology of the Jianping Complex: implications for the Precambrian crustal evolution history of the northern margin of North China

Craton. *Gondwana Research* 2011, **20**(1)**:** 48-63.

111. Becker RH, Clayton RN. Carbon isotopic evidence for the origin of a banded iron-formation in Western Australia. *Geochimica et Cosmochimica Acta* 1972, **36**(5)**:** 577-595.

112. Morris RC. Genetic modelling for banded iron-formation of the Hamersley Group, Pilbara Craton, Western Australia. *Precambrian research* 1993, **60**(1–4)**:** 243-286.

113. Wingate M. A palaeomagnetic test of the Kaapvaal-Pilbara (Vaalbara) connection at 2.78 Ga. *South African Journal of Geology* 1998, **101**(4)**:** 257-274.

114. Manikyamba C, Balaram V, Naqvi SM. Geochemical signatures of polygenetic origin of a banded iron formation (BIF) of the Archaean Sandur greenstone belt (schist belt) Karnataka nucleus, India. *Precambrian research* 1993, **61**(1–2)**:** 137-164.

115. Dalstra H, Bloem E, Ridley J, Groves D. Diapirism synchronous with regional deformation and gold mineralisation, a new concept for granitoid emplacement in the Southern Cross Province, Western Australia. *Geologie en Mijnbouw* 1998, **76**(4)**:** 321-338.

116. Qiu Y, McNaughton N, Groves D, Dalstra H. Ages of internal granitoids in the Southern Cross region, Yilgarn Craton, Western Australia, and their crustal evolution and tectonic implications*. *Australian Journal of Earth Sciences* 1999, **46**(6)**:** 971-981.

117. Mueller AG, McNaughton NJ. U-Pb Ages Constraining Batholith Emplacement, Contact Metamorphism, and the Formation of Gold and W-Mo Skarns in the Southern Cross Area, Yilgarn Craton, Western Australia. *Economic Geology* 2000, **95**(6)**:** 1231-1257.

118. Angerer T, Kerrich R, Hagemann SG. Geochemistry of a komatiitic, boninitic, and tholeiitic basalt association in the Mesoarchean Koolyanobbing greenstone belt, Southern Cross Domain, Yilgarn craton: Implications for mantle sources and geodynamic setting of banded iron formation. *Precambrian research* 2013, **224:** 110-128.

119. Trendall A, De Laeter J, Nelson D, Mukhopadhyay D. A precise zircon U-Pb age for the base of the BIF of the Mulaingiri Formation,(Bababudan Group, Dharwar Supergroup) of the Karnataka Craton. *Geological Society of India Journal* 1997, **50:** 161-170.

120. Corfu F, Wood J. U-Pb zircon ages in supracrustal and plutonic rocks; North Spirit Lake area, northwestern Ontario. *Canadian Journal of Earth Sciences* 1986, **23**(7)**:** 967-977.

121. Ribeiro da Luz B, Crowley JK. Morphological and chemical evidence of stromatolitic deposits in the 2.75 Ga Carajás banded iron formation, Brazil. *Earth and Planetary Science Letters* 2012, **355–356:** 60-72.

122. Haugaard R, Frei R, Stendal H, Konhauser K. Petrology and geochemistry of the 2.9 Ga Itilliarsuk

banded iron formation and associated supracrustal rocks, West Greenland: Source characteristics and depositional environment. *Precambrian research* 2013, **229:** 150-176.

123. Armstrong RA, Compston W, Retief EA, Williams IS, Welke HJ. Zircon ion microprobe studies bearing on the age and evolution of the Witwatersrand triad. *Precambrian research* 1991, **53**(3–4)**:** 243-266.

124. Krapez B. Sequence stratigraphy of the Archaean supracrustal belts of the Pilbara Block, Western Australia. *Precambrian research* 1993, **60**(1–4)**:** 1-45.

125. Pidgeon RT, Wilde SA. The distribution of 3.0 Ga and 2.7 Ga volcanic episodes in the Yilgarn Craton of Western Australia. *Precambrian research* 1990, **48**(3)**:** 309-325.

126. Horwitz R, Pidgeon R. 3.1 Ga tuff from the Sholl Belt in the West Pilbara: further evidence for d diachronous volcanism in the Pilbara Craton of Western Australia. *Precambrian research* 1993, **60**(1)**:** 175-183.

127. Van Kranendonk MJ. *Geology of the North Shaw 1: 100 000 Sheets: Sheets 2755*. Geological Survey of Western Australia: Perth, 2000.

128. Bontognali TRR, Fischer WW, Föllmi KB. Siliciclastic associated banded iron formation from the 3.2 Ga Moodies Group, Barberton Greenstone Belt, South Africa. *Precambrian research* 2013, **226:** 116-124.

129. Peucat J, Bouhallier H, Fanning C, Jayananda M. Age of the Holenarsipur greenstone belt, relationships with the surrounding gneisses (Karnataka, South India). *The Journal of Geology* 1995, **103**(6)**:** 701-710.

130. Mukhopadhyay J, Beukes N, Armstrong R, Zimmermann U, Ghosh G, Medda R. Dating the oldest greenstone in India: a 3.51-Ga precise U-Pb SHRIMP zircon age for dacitic lava of the southern Iron Ore Group, Singhbhum craton. *The Journal of Geology* 2008, **116**(5)**:** 449-461.

131. Rosing MT. ^{13}C-depleted carbon microparticles in >3700-Ma sea-floor sedimentary rocks from West Greenland. *Science* 1999, **283**(5402)**:** 674-676.

132. Nutman AP, Friend CRL, Paxton S. Detrital zircon sedimentary provenance ages for the Eoarchaean Isua supracrustal belt southern West Greenland: Juxtaposition of an imbricated ca. 3700 Ma juvenile arc against an older complex with 3920–3760 Ma components. *Precambrian research* 2009, **172**(3–4)**:** 212-233.

133. Mloszewska AM, Pecoits E, Cates NL, Mojzsis SJ, O'Neil J, Robbins LJ*, et al.* The composition of Earth's oldest iron formations: The Nuvvuagittuq Supracrustal Belt (Québec, Canada). *Earth and Planetary Science Letters* 2012, **317–318:** 331-342.

134. Nutman AP, McGregor VR, Shiraishi K, Friend CRL, Bennett VC, Kinny PD. ⩾3850 Ma BIF and

mafic inclusions in the early Archaean Itsaq Gneiss Complex around Akilia, southern West Greenland? The difficulties of precise dating of zircon-free protoliths in migmatites. *Precambrian research* 2002, **117**(3–4)**:** 185-224.

135. An Z, Jiang G, Tong J, Tian L, Ye Q, Song H*, et al.* Stratigraphic position of the Ediacaran Miaohe biota and its constrains on the age of the upper Doushantuo $\delta^{13}C$ anomaly in the Yangtze Gorges area, South China. *Precambrian research* 2015, **271:** 243-253.

136. Kaufman AJ, Jiang G, Christie-Blick N, Banerjee DM, Rai V. Stable isotope record of the terminal Neoproterozoic Krol platform in the Lesser Himalayas of northern India. *Precambrian Research* 2006, **147**(1)**:** 156-185.

137. Kaufman AJ, Corsetti FA, Varni MA. The effect of rising atmospheric oxygen on carbon and sulfur isotope anomalies in the Neoproterozoic Johnnie Formation, Death Valley, USA. *Chemical Geology* 2007, **237**(1)**:** 47-63.

138. Macdonald FA, Prave AR, Petterson R, Smith EF, Pruss SB, Oates K*, et al.* The Laurentian record of Neoproterozoic glaciation, tectonism, and eukaryotic evolution in Death Valley, California. *Geological Society of America Bulletin* 2013, **125**(7-8)**:** 1203-1223.

139. James N, Wray J, Ginsburg R. Calcification of encrusting aragonitic algae (Peyssonneliaceae): implications for the origin of Late Paleozoic reefs and cements. *Journal of Sedimentary Research* 1988, **58**(2)**:** 291-303.

140. Trower EJ, Grotzinger JP. Sedimentology, diagenesis, and stratigraphic occurrence of giant ooids in the Ediacaran Rainstorm Member, Johnnie Formation, Death Valley region, California. *Precambrian Research* 2010, **180**(1)**:** 113-124.

141. Jiang G, Kaufman AJ, Christie-Blick N, Zhang S, Wu H. Carbon isotope variability across the Ediacaran Yangtze platform in South China: Implications for a large surface-to-deep ocean $\delta^{13}C$ gradient. *Earth and Planetary Science Letters* 2007, **261**(1)**:** 303-320.

142. Zhu M, Lu M, Zhang J, Zhao F, Li G, Aihua Y*, et al.* Carbon isotope chemostratigraphy and sedimentary facies evolution of the Ediacaran Doushantuo Formation in western Hubei, South China. *Precambrian Research* 2013, **225:** 7-28.

143. Jiang G, Christie‐Blick N, Kaufman AJ, Banerjee DM, Rai V. Carbonate platform growth and cyclicity at a terminal Proterozoic passive margin, Infra Krol Formation and Krol Group, Lesser Himalaya, India. *Sedimentology* 2003, **50**(5)**:** 921-952.

144. Ziegler A, McKerrow W. Silurian marine red beds. *American Journal of Science* 1975, **275**(1)**:** 31-56.

145. Kiipli E, Kallaste T, Kiipli T. Hematite and goethite in Telychian marine red beds of the East Baltic. *GFF* 2000, **122**(3)**:** 281-286.

146. Liu J, Wang Y, Zhang X, Rong J. Early Telychian (Silurian) marine siliciclastic red beds in the Eastern Yangtze Platform, South China: distribution pattern and controlling factors. *Canadian Journal of Earth Sciences* 2016, **53**(7)**:** 712-718.

147. Hasegawa T, Crampton JS, Schiøler P, Field B, Fukushi K, Kakizaki Y. Carbon isotope stratigraphy and depositional oxia through Cenomanian/Turonian boundary sequences (Upper Cretaceous) in New Zealand. *Cretaceous research* 2013, **40:** 61-80.

148. Bristow TF, Kennedy MJ. Carbon isotope excursions and the oxidant budget of the Ediacaran atmosphere and ocean. *Geology* 2008, **36**(11)**:** 863-866.

149. Rothman DH, Hayes JM, Summons RE. Dynamics of the Neoproterozoic carbon cycle. *Proceedings of the National Academy of Sciences* 2003, **100**(14)**:** 8124-8129.

150. Guerroué E, Allen PA, Cozzi A, Etienne JL, Fanning M. 50 Myr recovery from the largest negative $\delta^{13}C$ excursion in the Ediacaran ocean. *Terra Nova* 2006, **18**(2)**:** 147-153.

151. Halverson GP, Hoffman PF, Schrag DP, Maloof AC, Rice AHN. Toward a Neoproterozoic composite carbon-isotope record. *GSA Bulletin* 2005, **117:** 1181-1207.

152. Fike D, Grotzinger J, Pratt L, Summons R. Oxidation of the Ediacaran ocean. *Nature* 2006, **444**(7120)**:** 744-747.

153. Grotzinger JP, Fike DA, Fischer WW. Enigmatic origin of the largest-known carbon isotope excursion in Earth's history. *Nature Geoscience* 2011, **4**(5)**:** 285-292.

154. Husson JM, Maloof AC, Schoene B, Chen CY, Higgins JA. Stratigraphic expression of Earth's deepest $\delta^{13}C$ excursion in the Wonoka Formation of South Australia. *American Journal of Science* 2015, **315**(1)**:** 1-45.

155. Knoll A, Hayes J, Kaufman A, Swett K, Lambert I. Secular variation in carbon isotope ratios from Upper Proterozoic successions of Svalbard and East Greenland. *Nature* 1986, **321:** 832-838.

156. Grotzinger JP, Knoll AH. Anomalous carbonate precipitates; is the Precambrian the key to the Permian? *Palaios* 1995, **10**(6)**:** 578-596.

157. McFadden KA, Huang J, Chu X, Jiang G, Kaufman AJ, Zhou C*, et al.* Pulsed oxidation and biological evolution in the Ediacaran Doushantuo Formation. *Proceedings of the National Academy of Sciences* 2008, **105**(9)**:** 3197-3202.

158. Johnston DT, Macdonald FA, Gill B, Hoffman P, Schrag DP. Uncovering the Neoproterozoic carbon cycle. *Nature* 2012, **483**(7389)**:** 320-323.

159. Jiang G, Wang X, Shi X, Xiao S, Zhang S, Dong J. The origin of decoupled carbonate and organic carbon isotope signatures in the early Cambrian (ca. 542–520 Ma) Yangtze platform. *Earth and Planetary Science Letters* 2012, **317:** 96-110.

160. Jiang G, Wang X, Shi X, Zhang S, Xiao S, Dong J. Organic carbon isotope constraints on the dissolved organic carbon (DOC) reservoir at the Cryogenian–Ediacaran transition. *Earth and Planetary Science Letters* 2010, **299**(1)**:** 159-168.

161. Wang X, Jiang G, Shi X, Xiao S. Paired carbonate and organic carbon isotope variations of the Ediacaran Doushantuo Formation from an upper slope section at Siduping, South China. *Precambrian Research* 2016, **273:** 53-66.

162. Swart P, Kennedy M. Does the global stratigraphic reproducibility of $\delta^{13}C$ in Neoproterozoic carbonates require a marine origin? A Pliocene–Pleistocene comparison. *Geology* 2012, **40**(1)**:** 87-90.

163. Knauth LP, Kennedy MJ. The late Precambrian greening of the Earth. *Nature* 2009, **460**(7256)**:** 728-732.

164. Derry LA. A burial diagenesis origin for the Ediacaran Shuram–Wonoka carbon isotope anomaly. *Earth and Planetary Science Letters* 2010, **294**(1)**:** 152-162.

165. Schrag DP, Higgins JA, Macdonald FA, Johnston DT. Authigenic carbonate and the history of the global carbon cycle. *Science* 2013, **339**(6119)**:** 540-543.

166. Cui H, Kaufman AJ, Xiao S, Zhou C, Liu X-M. Was the Ediacaran Shuram Excursion a globally synchronized early diagenetic event? Insights from methane-derived authigenic carbonates in the uppermost Doushantuo Formation, South China. *Chemical Geology* 2017, **450:** 59-80.

167. Tsikos H, Karakitsios V, Van Breugel Y, Walsworth-Bell B, Bombardiere L, Petrizzo MR*, et al.* Organic-carbon deposition in the Cretaceous of the Ionian Basin, NW Greece: the Paquier Event (OAE 1b) revisited. *Geological Magazine* 2004, **141**(04)**:** 401-416.

168. Stoll HM, Schrag DP. Sr/Ca variations in Cretaceous carbonates; relation to productivity and sea level changes. *Palaeogeography, Palaeoclimatology, Palaeoecology* 2001, **168:** 311-336.

169. Wendler I, Wendler J, Neuhuber S, Wagreich M. Productivity fluctuations and orbital cyclicity during onset of Early to Middle Turonian marine red-bed formation (Austrian Eastern Alps). *SEPM Special Publication* 2009, **91:** 209-221.

170. Jarvis I, Gale AS, Jenkyns HC, Pearce MA. Secular variation in Late Cretaceous carbon isotopes: a new $\delta^{13}C$ carbonate reference curve for the Cenomanian–Campanian (99.6–70.6 Ma). *Geological Magazine* 2006, **143**(05)**:** 561-608.

171. Stein M, Föllmi KB, Westermann S, Godet A, Adatte T, Matera V*, et al.* Progressive

palaeoenvironmental change during the late Barremian–early Aptian as prelude to Oceanic Anoxic Event 1a: Evidence from the Gorgo a Cerbara section (Umbria-Marche basin, central Italy). *Palaeogeography, Palaeoclimatology, Palaeoecology* 2011, **302**(3)**:** 396-406.

172. Hu X, Scott RW, Cai Y, Wang C, Melinte-Dobrinescu MC. Cretaceous oceanic red beds (CORBs): Different time scales and models of origin. *Earth-Science Reviews* 2012, **115**(4)**:** 217-248.

173. O'Dogherty L, Sandoval J, Bartolini A, Bruchez S, Bill M, Guex J. Carbon isotope stratigraphy and ammonite faunal turnover for the Middle Jurassic in the Southern Iberian palaeomargin. *Palaeogeography, Palaeoclimatology, Palaeoecology* 2006, **239**(3)**:** 311-333.

174. Bachan A, Schootbrugge B, Fiebig J, McRoberts CA, Ciarapica G, Payne JL. Carbon cycle dynamics following the end‐Triassic mass extinction: Constraints from paired δ^{13}Ccarb and δ^{13}Corg records. *Geochemistry, Geophysics, Geosystems* 2012, **13**(9)**:** 1-24.

175. Wotte T, Strauss H, Fugmann A, Garbe-Schönberg D. Paired δ^{34}S data from carbonate-associated sulfate and chromium-reducible sulfur across the traditional Lower–Middle Cambrian boundary of W-Gondwana. *Geochimica et Cosmochimica Acta* 2012, **85:** 228-253.

176. Brasier M, Khomentovsky V, Corfield R. Stable isotopic calibration of the earliest skeletal fossil assemblages in eastern Siberia (Precambrian‐Cambrian boundary). *Terra Nova* 1993, **5**(3)**:** 225-232.

177. Winter BL, Knauth LP. Stable isotope geochemistry of cherts and carbonates from the 2.0 Ga Gunflint Iron Formation: implications for the depositional setting, and the effects of diagenesis and metamorphism. *Precambrian Research* 1992, **59**(3)**:** 283-313.

178. Beukes NJ, Klein C, Kaufman AJ, Hayes J. Carbonate petrography, kerogen distribution, and carbon and oxygen isotope variations in an early Proterozoic transition from limestone to iron-formation deposition, Transvaal Supergroup, South Africa. *Economic geology and the bulletin of the Society of Economic Geologists* 1990, **85**(4)**:** 663-690.

179. Baur M, Hayes J, Studley S, Walter M. Millimeter-scale variations of stable isotope abundances in carbonates from banded iron-formations in the Hamersley Group of Western Australia. *Economic Geology* 1985, **80**(2)**:** 270-282.

180. Perry E, Tan F. Significance of oxygen and carbon isotope variations in early Precambrian cherts and carbonate rocks of southern Africa. *Geological Society of America Bulletin* 1972, **83**(3)**:** 647-664.

181. Craddock PR, Dauphas N. Iron and carbon isotope evidence for microbial iron respiration throughout the Archean. *Earth and Planetary Science Letters* 2011, **303**(1)**:** 121-132.
